# Short term starvation potentiates the efficacy of chemotherapy in triple negative breast cancer via metabolic reprogramming

**DOI:** 10.1186/s12967-023-03935-9

**Published:** 2023-03-03

**Authors:** Ioannis S. Pateras, Chloe Williams, Despoina D. Gianniou, Aggelos T. Margetis, Margaritis Avgeris, Pantelis Rousakis, Aigli-Ioanna Legaki, Peter Mirtschink, Wei Zhang, Konstantina Panoutsopoulou, Anastasios D. Delis, Stamatis N. Pagakis, Wei Tang, Stefan Ambs, Ulrika Warpman Berglund, Thomas Helleday, Anastasia Varvarigou, Antonios Chatzigeorgiou, Anders Nordström, Ourania E. Tsitsilonis, Ioannis P. Trougakos, Jonathan D. Gilthorpe, Teresa Frisan

**Affiliations:** 1 2nd Department of Pathology, “Attikon” University Hospital, Medical School, National and Kapodistrian University of Athens, 124 62 Athens, Greece; 2 Department of Integrative Medical Biology, Umeå University, 901 87 Umeå, Sweden; 3 Department of Cell Biology and Biophysics, Faculty of Biology, National and Kapodistrian University of Athens, 157 84 Athens, Greece; 4 2nd Department of Internal Medicine, Athens Naval and Veterans Hospital, 115 21 Athens, Greece; 5 Laboratory of Clinical Biochemistry-Molecular Diagnostics, Second Department of Pediatrics, School of Medicine, National and Kapodistrian University of Athens, “P. & A. Kyriakou” Children’s Hospital, 115 27 Athens, Greece; 6 Department of Biochemistry and Molecular Biology, Faculty of Biology, National and Kapodistrian University of Athens, 157 71 Athens, Greece; 7 Department of Biology, School of Science, National and Kapodistrian University of Athens, 157 84 Athens, Greece; 8 Department of Physiology, Medical School, National and Kapodistrian University of Athens, 115 27 Athens, Greece; 9 Institute for Clinical Chemistry and Laboratory Medicine, University Hospital and Faculty of Medicine, Technische Universität Dresden, 013 07 Dresden, Germany; 10 Swedish Metabolomics Centre, Department of Plant Physiology, Umeå University, 901 87 Umeå, Sweden; 11 Centre for Basic Research, Bioimaging Unit, Biomedical Research Foundation, Academy of Athens, 115 27 Athens, Greece; 12 Molecular Epidemiology Section, Laboratory of Human Carcinogenesis, Center for Cancer Research (CCR), NCI, NIH, Bethesda, MD 20892-4258 USA; 13 Science for Life Laboratory, Department of Oncology-Pathology, Karolinska Institutet, 171 76 Stockholm, Sweden; 14 Weston Park Cancer Centre, Department of Oncology and Metabolism, University of Sheffield, Sheffield, S10 2RX UK; 15 Department of Paediatrics, University of Patras Medical School, General University Hospital, 265 04 Patras, Greece; 16 Department of Molecular Biology and Umeå Centre for Microbial Research (UCMR), Umeå University, 901 87 Umeå, Sweden; 17 Data Science & Artificial Intelligence, R&D, AstraZeneca, Gaithersburg, MD USA

**Keywords:** Breast cancer, Triple negative breast cancer, Caloric restriction, Fasting, Starvation, Oxidative stress, Reactive oxygen species, Mitochondria, Metabolic reprogramming, Oncological treatment

## Abstract

**Background:**

Chemotherapy (CT) is central to the treatment of triple negative breast cancer (TNBC), but drug toxicity and resistance place strong restrictions on treatment regimes. Fasting sensitizes cancer cells to a range of chemotherapeutic agents and also ameliorates CT-associated adverse effects. However, the molecular mechanism(s) by which fasting, or short-term starvation (STS), improves the efficacy of CT is poorly characterized.

**Methods:**

The differential responses of breast cancer or near normal cell lines to combined STS and CT were assessed by cellular viability and integrity assays (Hoechst and PI staining, MTT or H_2_DCFDA staining, immunofluorescence), metabolic profiling (Seahorse analysis, metabolomics), gene expression (quantitative real-time PCR) and iRNA-mediated silencing. The clinical significance of the in vitro data was evaluated by bioinformatical integration of transcriptomic data from patient data bases: The Cancer Genome Atlas (TCGA), European Genome-phenome Archive (EGA), Gene Expression Omnibus (GEO) and a TNBC cohort. We further examined the translatability of our findings in vivo by establishing a murine syngeneic orthotopic mammary tumor-bearing model.

**Results:**

We provide mechanistic insights into how preconditioning with STS enhances the susceptibility of breast cancer cells to CT. We showed that combined STS and CT enhanced cell death and increased reactive oxygen species (ROS) levels, in association with higher levels of DNA damage and decreased mRNA levels for the NRF2 targets genes *NQO1* and *TXNRD1* in TNBC cells compared to near normal cells. ROS enhancement was associated with compromised mitochondrial respiration and changes in the metabolic profile, which have a significant clinical prognostic and predictive value. Furthermore, we validate the safety and efficacy of combined periodic hypocaloric diet and CT in a TNBC mouse model.

**Conclusions:**

Our in vitro, in vivo and clinical findings provide a robust rationale for clinical trials on the therapeutic benefit of short-term caloric restriction as an adjuvant to CT in triple breast cancer treatment.

**Supplementary Information:**

The online version contains supplementary material available at 10.1186/s12967-023-03935-9.

## Background

Breast cancer is the most common malignancy among women worldwide, with 2,000,000 new cases annually. Triple negative breast cancer (TNBC) accounts for almost 15% of all diagnosed breast cancer cases and is an aggressive cancer with the poorest prognosis among all breast cancer subtypes [1]. Chemotherapy (CT) including anthracycline-based regimes remains the treatment of choice for TNBC, however severe toxic effects can limit their application [2]. Therefore, it is important to develop effective therapeutic approaches that selectively target cancer cells and protect normal cells against the cytotoxic effect of CT [3, 4].

A growing body of evidence supports the hypothesis that fasting enhances the efficacy of chemotherapeutic agents in various types of cancer, while protecting normal cells, suggesting a model of differential stress resistance (DSR) [5–7]. Cycles of short-term starvation (STS) have also been found to sensitize cancer cell lines to a range of chemotherapeutic agents and promote survival rates in mouse cancer models [8–10]. Importantly, fasting mice treated with high doses CT do not exhibit signs of toxicity, suggesting that STS can ameliorate the detrimental side effects of CT in normal cells [8–10]. These data suggest that cancer cells are unable to respond appropriately in terms of their metabolic response to STS, which acts to protect normal cells. From a clinical perspective complete abstinence from food could lead to other effects, such as unacceptable weight loss that should be avoided, especially in patients developing cachexia [11]. Therefore, various other strategies are under investigation, including periodic dieting that mimics fasting [12, 13], short-term fasting [14] and mimetics of caloric restriction [15] which could be more suited to clinical use. In order to implement safe and effective dietary modifications into cancer therapy, it is first necessary to better understand the molecular mechanisms that promote the specific enhanced sensitivity of starved cancer cells to CT.

Here, we provide novel insights showing that the increased susceptibility to CT by STS in triple negative and luminal type breast cancer cell lines is achieved by altering their redox status and promoting reactive oxygen species (ROS)-induced cytotoxicity. This effect is mediated, at least in part, through metabolic rewiring and enhanced mitochondrial dysfunctionality that has a high prognostic value. Conversely, non-transformed mammary epithelial cells were protected from CT-induced cell death upon preconditioning with STS, which further promoted their ability to counteract ROS.

In a syngeneic orthotopic triple negative mammary cancer mouse model, cycles of short-term caloric restriction together with CT had a combined effect, resulting in significant inhibition of breast tumor growth and metastasis. Importantly this approach was safe with no signs of significant weight loss or systemic toxicity.

Our data highlight key molecular mechanisms that help to understand the enhanced sensitivity of cancer cells to starvation and furthermore show that, within breast cancer subtypes, TNBC may be susceptible to this type of intervention, providing rationale for the development of additional therapeutic approaches.

## Methods

### Cell culture

Human malignant breast cell lines MCF-7 (ATCC HTB-22), SKBR-3 (ATCC HTB-30), MDA-MB-231 (ATCC CRM-HTB-26), MDA-MB-468 (ATCC HTB-132) and HS578 (HS578T, ATCC HTB-126) were provided by Federica Cavalo (University of Turin, Molecular Biotechnology Center, Turin, Italy), Constantin N. Baxevanis (“Saint Savvas” Cancer Hospital Athens, Greece) and Apostolos Klinakis (Center for Basic Research, Biomedical Research Foundation of the Academy of Athens, Athens, Greece). The near normal mammary human epithelial cell line MCF-10A cell line (ATCC CRL10317) was provided by Anastasios Papanastasiou (University of Patras, Medical School, Patra, Greece). The mouse malignant cell line 4T1 (ATCC CRL-2539) was provided by Konstantinos Dimas (University of Thessaly, Medical School, Larisa, Greece). MDA-MB-231, MDA-MB-468, HS578 and MCF-7 cells were cultured in DMEM (Thermo Fischer Scientific, Waltham, MA, USA) containing 2 g/L glucose, 10% FBS (Thermo Fischer Scientific) and 1% penicillin/streptomycin (Sigma-Aldrich, St. Louis, MO, USA) at 37 °C in a 5% CO_2_ incubator. SKBR-3 and 4T1 cells were cultured in RPMI (Thermo Fischer Scientific) containing 4 g/L glucose, 10% FBS, 1% penicillin/streptomycin. Medium was renewed every 48–72 h. MCF-10A cells were grown in DMEM/HAM’s F12 medium (Biochrom) supplemented with 5% horse serum (Thermo Fischer Scientific), 10 μg/ml insulin (Sigma-Aldrich), 20 ng/ml EGF (Peprotech), 0.5 μg/ml hydrocortisone (Sigma-Aldrich), 100 ng/ml cholera toxin (Sigma-Aldrich) and 1%penicillin/streptomycin. The culture medium is defined as complete medium.

Treatment with short-term starvation was started at 70–80% cell confluence. STS medium consisted of DMEM containing 1 g/L glucose, 2% FBS, 1% penicillin/streptomycin for the MCF-7, MDA-MB-231 and MDA-MB-468, HS578 cell lines; RPMI containing 2 g/L glucose, 2% FBS, 1% penicillin/streptomycin for the SKBR-3 and 4T1 cell lines [9, 16]; DMEM/HAM’s F12 medium supplemented with 5 μg/ml insulin, 4 ng/ml EGF, 0.5 μg/ml hydrocortisone, 100 ng/ml cholera toxin, 1% penicillin/streptomycin, and 1% horse serum for the MCF-10A cells [9, 16].

STS treatment was performed for 48 h and chemotherapeutic agents were added for the subsequent 24 h at the indicated concentrations in starvation medium (Additional file 1: Fig. S1a) [17].

### Metabolomics and survival correlation in the TNBC cohort

Metabolomic profiling of human breast tissues was performed using an untargeted approach. Untargeted metabolic profiling of known and unknown metabolites was performed in 67 human breast tumors and 65 tumor-adjacent noncancerous tissues by Metabolon Inc, as described previously [18, 19]. The TNBC cohort includes patients recruited in Baltimore (Maryland, USA) hospitals between 1993 and 2003, as previously described [18, 19]. Clinical and pathological information (e. g. hormone receptor status) was obtained from medical records and pathology reports. Triple-negative tumors were negative for estrogen, progesterone, and HER2 receptor expression. In total 17 triple-negative tumors where the metabolic analysis was available were identified [18, 19]. Survival analysis was performed in these 17 patients, who had long-term follow up for breast cancer-specific survival.

### Chemicals and pharmaceuticals agents

Cisplatin (1 mg/mg solution for injection, Pfizer Hellas S.A.) and doxorubicin hydrochloride (2 mg/ml, Pfizer Hellas S.A.) were diluted in DMEM before usage in vitro; TH1579 (karonudib 10 mM provided by Thomas Helleday Foundation), and gemcitabine (Sigma-Aldrich) were diluted in DMSO before usage. N-acetylcysteine (NAC, A7250 Sigma-Aldrich) was diluted at 100 mg/ml in phosphate buffered saline (PBS), filtered, pH-tested and aliquots stored at − 20 °C.

### MTT assay

Cells were washed once with PBS and fresh medium supplemented with the MTT solution (Sigma-Aldrich) (10 μl of the 5 mg/ml MTT solution in 100 μl medium for each well). Cells were incubated in the dark at 37 °C for 6 h to allow the formation of purple-black formazan crystals. Medium was replaced with DMSO (Sigma-Aldrich) and absorbance was recorded in a plate reader at 650 nm and 570 nm. Background optical density (650 nm) was subtracted from the 570 nm absorbance values as previously described [20].

### Intracellular ROS measurement

For the assessment of ROS production, cells were incubated with 10 μM CM-2',7'-dichlorodihydrofluorescein diacetate (H_2_DCFDA, Thermo Fisher Scientific) in PBS for 30 min at 37 °C in the dark. Following dye removal, cells were incubated for 10 min with PBS and lysed in Nonidet P-40 lysis buffer (1% Nonidet P-40, 150 mM NaCl, and 50 mM Tris, pH 8.0). Lysates were cleared by centrifugation at 19,000*g* for 10 min at 4 °C. The supernatant was diluted 1:4 (v/v) in ddH_2_O, and fluorescence was measured using a VersaFluor Fluorometer System (Bio-Rad Laboratories, Hercules, CA, USA) at 490 nm excitation and 520 nm emission.

### Hoechst 33342 and propidium iodide (PI) staining

Depending on cell type, 5,000–10,000 cells/well were seeded in black sided 96-well plates (Cell Star, uClear, Greiner Bio-One, Frickenhausen, Germany), and the STS or STS-DXR treatment was performed. At the endpoint, Hoechst 33342 and PI stain were diluted in Opti-MEM (Gibco) at a final concentration of 5 μg/ml for each compound and 100 μl added per well. Cells were incubated at 37 °C for 5 min before the addition of 200 μl Opti-MEM to fill the well. The fluorescent signals for Hoechst 33342 and PI were measured at 405/450 and 540/640 (nm excitation/emission), respectively, using a microplate imager (PlateRUNNER HD, TROPHOS, Marseille, France).

### Silencing treatment

MCF10A, MDA-MB-231, MDA-MB-468 and HS578 cells were seeded at a density of 30,000 cells/cm^2^ in 6-well tissue culture plates. Twenty-four hours later the cells were transfected with 50 nM of the SMARTpool ON-TARGET plus FOXO1 siRNA (L-003006–00-0005), the SMARTpool ON-TARGET plus ATP5A1 siRNA (L-017064–01-0005) or the ON-TARGETplus non-targeting pool (Scramble) (D-001810–10-05) (GE Healthcare Dharmacon Inc.). All transfections were carried out using DharmaFECT Transfection reagent (T-2001–03) (GE Healthcare Dharmacon Inc.), according to manufacturer’s instructions. siRNAs and DharmaFECT Transfection reagent were diluted in Opti-MEM I (31,985,062, Gibco). Transfected cells were plated in 96 well plates and grown in the same complete STS or STS + DXR mediums as previously described. Gene silencing results were validated by Q-RT-PCR.

### Seahorse cellular stress assays

To evaluate the changes in mitochondrial and glycolytic function Seahorse XFp Cell MitoStress Test and Seahorse XFp Glycolysis Stress Test (Agilent Technologies, Santa Clara, CA) were performed according to the manufacturer’s protocol. In brief, MCF10A or MDA-MB-231 cells were plated at 40,000 cells/well in poly-D-lysine (50 µg/ml, Sigma-Aldrich) coated plates. The same number of cells were plated for each cell line. On the day of the assay the cell culture growth medium was replaced with either MitoStress or GlycoStress assay medium. MitoStress assay medium consisted of low-buffered pH 7.4 DMEM (Sigma-Aldrich) supplemented with glutamine (2 mM, ThermoFisher Scientific), glucose (10 mM, ThermoFisher Scientific) and pyruvate (1 mM, ThermoFisher Scientific). GlycoStress assay medium consisted of low-buffered pH 7.4 DMEM supplemented with glutamine (2 mM). The cell culture microplate was incubated in a non-CO2 incubator at 37 °C for 1 h prior to the assay. The seahorse compounds were prepared in assay media and injected into the injection ports.

For MitoStress assays, oligomycin (1 µM, Sigma-Aldrich), carbonyl cyanide 4-(trifluoromethoxy) phenylhydrazone (FCCP, 2 µM, Sigma-Aldrich) and rotenone/antimycin A (0.5 µM each, Sigma-Aldrich) were used. For glycolysis stress assays, glucose (10 mM), oligomycin (1 µM) and 2-deoxy glucose (50 mM, Sigma-Aldrich) were used.

Oxygen consumption rate (OCR) and extracellular acidification rate (ECAR) were reported as absolute rates (pmoles/min for OCR and mpH/min for ECAR). Data were exported from the Seahorse XFp Extracellular Flux Analyser into Seahorse XF Report Generator software. The nine replicates for each condition (3 technical replicates for each of the 3 biological replicates) were compiled in GraphPad Prism 7 Software.

### LC/MS analysis

Metabolic profiling by LC–MS was performed at the Swedish Metabolomics Center (Umeå University, Umeå, Sweden). Further details are included in Additional file 8.

### RNA isolation and quantitative real time PCR (q-RT-PCR) analysis

Total RNA was isolated using the NucleoZOL RNA Isolation Reagent (Macherey–Nagel, Düren, Germany) and quantified with BioSpec-nano spectrophotometer (Shimadzu Inc.). Subsequently, cDNA synthesis and qRT-PCR were performed using the FastGene Scriptase II cDNA Synthesis 5 × Ready-Mix (NIPPON Genetics EUROPE, GmbH, Düren, Germany) and the KAPA SYBR FAST qPCR Master Mix (2X), respectively. Primers were designed using the primer-BLAST tool (http://www.ncbi.nlm.nih.gov/tools/primer-blast/). Further details for the q-RT-PCR analysis are provided in [21].

### Histopathological evaluation

The histopathological evaluation was performed on hematoxylin–eosin (H&E) stained sections with 2.5 μm thickness. The number of mitotic cells per high power field (HPF, 400 × magnification) were evaluated in the primary lesions. For the evaluation of the metastatic lesions, the total number of metastatic lesions with > 150 μm maximum diameter in liver and lung was assessed.

### Immunofluorescence analysis

Coverslips from culture dishes were fixed in 4% paraformaldehyde, rinsed and stored in PBS at 4 °C. Fixed cells were permeabilized with permeabilization and blocking buffer (Triton-X 0.3%, bovine serum albumin, BSA, 1% and goat serum) for 30 min. Mouse anti-ATP5A (Abcam, ab14748, diluted 1:200 in blocking buffer) or anti-γH2AX (#9718, Cell Signaling Technology, MA, USA, diluted 1:100) primary antibody was added overnight at 4 °C. Slides were washed twice in PBS. Secondary *Alexa Fluor*® 488-conjugated anti-mouse secondary antibody (Invitrogen, Waltham, MA USA, diluted 1:400 in blocking buffer) was added for 1 h incubation at 20 °C. Nuclei were counterstained with DAPI (Thermo Fisher Scientific). Each staining was performed in at least 3 biological triplicates. Samples were viewed with a NIKON C1 Confocal Laser Scanning Microscope (CLSM). Quantification of the ATP5A fluorescence staining (CTCF; corrected total cell fluorescence) was performed in ImageJ Software as previously described (https://theolb.readthedocs.io/en/latest/imaging/measuring-cell-fluorescence-using-imagej.html). Briefly, integrated density was measured for each cell (at least 15 cells/treatment group) and nearby background. CTCF for each cell was calculated according to the formula: CTCF = Integrated density – (area of selected cell x mean fluorescence of background readings). Plots represent average CTCF for each treatment group. For quantification of γH2AX fluorescence staining, we counted the number of γH2AX foci per cell, per high power field (magnification, 400×).

### In vivo study

Male and female BALB/c mice of 4 weeks old were purchased from Harlan Laboratories (Udine, Italy) and housed under standard laboratory conditions (temperature 22 °C; humidity 55 ± 10%; photoperiod 12 h light, 12 h dark) in a pathogen-free unit. After 3 weeks of acclimation, the daily intake of regular chow (vacuum-packed pelleted rodent chow containing 18.5% protein, 5.5% fat, 4.5% fiber, 6% ash; 4RF22, Mucedola, Milan, Italy) by individually caged mice was determined by serial measurements after giving defined amount of food. Consistent with previous reports and considering mouse weight, we estimated that food consumption of male approximates 3.9 g/day and female mice 3.2 g/day. Hence, male and female mice in caloric restriction (CR) groups were offered 2.73 g and 2.24 g food pellets, respectively, in a daily aliquot. Food consumption ad libitum was continuously monitored over the course of the experiment to ensure adequate nourishment and be aware of any adjustments needed to be considered for CR groups. After 3 days on CR diet, mice ate the whole amount of their food daily. Weight and general appearance were routinely monitored, and animals were euthanized if lethargic or if weight loss exceeded 20%. To establish the orthotopic breast cancer model, 1 × 10^5^ 4T1 cells, suspended in PBS/trypan blue, were subcutaneously injected in one inguinal mammary fat pad of each mouse after intraperitoneal (i.e.) anesthesia with ketamine. Tumor growth was measured every second day (4 days/week) with a digital caliper and tumor volume was calculated using the formula V = (L*W*H)/2 (L = length, W = width, H = height). The treatment protocol started 13 days post-implantation when average tumor volume was 75–85 mm^3^. Mice were randomized into 4 subgroups: control (N = 6), doxorubicin (N = 4), caloric restriction (N = 5), caloric restriction + doxorubicin (N = 5). For drug treatment, doxorubicin hydrochloride 2 mg/ml (Pfizer Hellas S.A.) was administered (5 mg/kg) on days 4, 11 and 18, after starting caloric restriction (CR), as i.p. bolus in 100 μL saline. Control/CR mice were given 100μL saline i.p. bolus. Thirty-five days post-injection, 200 μl blood was drawn from the tail vein for each animal and mice were euthanized by cervical dislocation and autopsied. Blood was centrifuged and serum was stored ice-cold. Serum was subjected to biochemical analysis. A small piece of the primary tumor was collected for mitochondrial isolation and measurement of mitochondrial respiration ex vivo and the remaining was formalin-fixed for histopathological evaluation. Liver and lung were collected from all animals in formalin medium for fixation and were microscopically investigated for metastatic foci or other signs of pathology.

### Biochemical analysis

The activity of aspartate- and alanine-amino transferase (ASAT and ALAT respectively), creatine kinase (CK) and lactate dehydrogenase (LDH), as well as the levels of glucose, albumin, urea, creatinine, and potassium in mouse serum were measured in a Cobas 8000 Analyzer (Roche, Basel, Switzerland).

### Mitochondria isolation and measurement of mitochondrial respiration ex vivo

Mitochondria were isolated as previously described [22]. Briefly tumors were homogenized in ice-cold isolation buffer (0.32 M sucrose, 10 mM EDTA, 10 mM Tris/HCl, pH 7.3) containing 2% BSA. Samples were filtered through a layer of gauze which was washed with additional isolation buffer up to a final volume of 1.5 ml. Following centrifugation for 10 min at 2,200 g, the pellet was washed with BSA-free isolation buffer and resuspended in 200 μl of the same buffer. Protein content of isolated mitochondria was assayed by Bradford assay (Thermo Fisher Scientific).

Mitochondrial respiration was determined using a Clark-type O_2_ electrode connected to a computer operated Oxygraph control unit (Hansatech Instruments, Norfolk, U.K.) as previously described [23]. Freshly isolated mitochondria (150 μg of protein) were added to the respiration buffer (120 mM KCl, 5 mM KH_2_PO_4_, 3 mM HEPES, 1 mM EGTA, 1 mM MgCl_2_, 0.2% BSA, pH 7.2) containing 5 mM glutamate/2.5 mM malate. Basal O_2_ consumption was recorded (state 2) and after 2 min 500 μM ADP was added (State 3; which measures rate of ATP production, O_2_ consumption), followed by 6 μΜ oligomycin (State 4, which measures coupling) and 100 nM of the uncoupler FCCP (to measure maximal respiration; State FCCP). In all experiments, the temperature was maintained at 25 °C and the total reaction volume was 300 μl. The respiratory control ratio (RCR) was calculated as the ratio of State 3 to State 4 (ST3/ST4).

### Bioinformatic analysis

The association between the expression of different genes with survival employing the Kaplan–Meier plotter for breast cancer was assessed using an open-source web software (https://kmplot.com/analysis/). The software includes mRNA data by GEO, EGA, and TCGA [24] [25].

The open source software ROC plotter for breast cancer [26] was used to link the transcription expression of different genes to anthracycline response.

### Statistics

Statistical analysis was performed by IBM SPSS Statistics 20 software (IBM Corp., Armonk, New York, USA). Independent samples *t*-test and one-way ANOVA were applied for the comparison of means between 2 and ≥ 3 groups, respectively. A sample size of n ≥ 3 was used for each sample group in a given experiment. Data are presented as dot-plots, box-plots, whisker-plots or heatmaps. For metabolomics and gene expression analysis, the heat maps and hierarchical clustering were generated using the ComplexHeat map R package and GraphPad Prism 7 [27]. For survival analysis of the 17 patients of the TNBC cohort, the Cox Proportional-Hazards Regression model was applied to estimate hazard ratios (HRs) and a Wald test was used to evaluate the significance of outcome differences between risk groups. In the analysis, tumor metabolite levels were generally median-dichotomized to define high-abundance and low-abundance groups in the Metabolon dataset. Survival analyses were conducted using R (R Foundation for Statistical Computing; http://www.r-project.org/). All statistical tests were two-sided. P < 0.05 was considered statistically significant.

## Results

### Starvation preferentially sensitizes triple negative breast cancer cells to chemotherapy compared to near normal cells

**Fig. 1:**
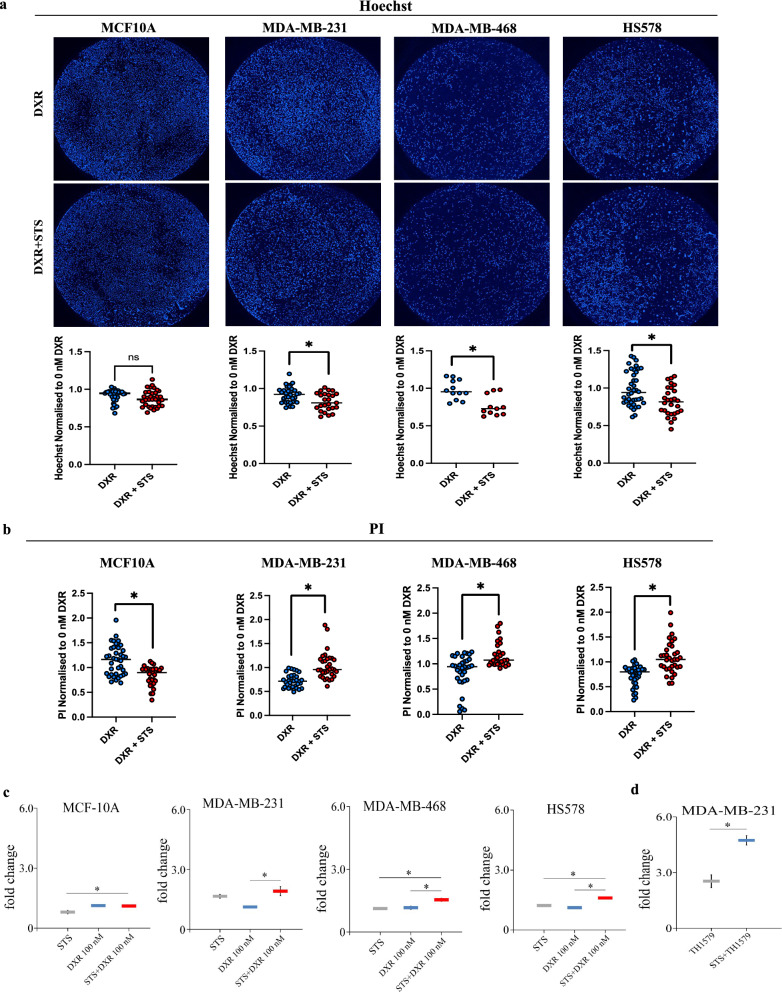
Differential sensitivity of cancer cells to STS and DXR is associated with enhanced ROS production in triple negative breast cancer cells. **a** Starvation in combination with 100 nM DXR treatment selectively inhibits cellular proliferation (Hoechst) in MDA-MB-231, MDA-MB-468 and HS578 cancer cells but not in the near normal MCF-10 cell line. Scale bar: 1 mm. **b** Starvation in combination with 100 nM DXR treatment selectively increases cell death (PI) in MDA-MB-231, MDA-MB-468 and HS578 cancer cells but not in the near normal MCF-10 cell line. **c** Assessment of intracellular ROS production. Data are presented as mean of the fold change ± SD of intracellular ROS production in MDA-MB-231, MDA-MB-468 and HS578 and MCF-10A cells upon STS, DXR or combined STS + DXR treatment. *P ≤ 0.05. **d** Assessment of intracellular ROS production. Data are presented as mean of the fold change ± SD in MDA-MB-231 cells treated with STS with or without the MTH1 inhibitor TH1579. *P ≤ 0.05

The aggressive TNBC cell lines MDA-MB-231, MDA-MB-468 and HS578 and the near normal human epithelial mammary cell line MCF-10A [28] were compared for their responses to 100 nM doxorubicin (DXR, an anthracycline class medication) with or without prior serum starvation (Additional file 1: Fig. S1a). STS potentiated the cytotoxic effects of DXR in the TNBC cell lines, as assessed by reduced cell recovery (Hoechst staining) and increased cell death (permeability to PI) (Fig. 1a, b). Conversely a reduced level of cell death was observed in the near normal MCF-10A cells (Fig. 1a, b). The cytotoxic effect of the combined treatment in breast cancer cells was validated by the MTT assay in the TNBC cell lines (Additional file 1: Fig. S1c) and was further extended to the MCF7 (luminal type A) and SKBR-3 (HER2 positive) breast cancer cell lines, representing clinically and pathologically distinct forms of breast carcinoma (Additional file 1: Fig. S1b). Although still responsive to CT, SKBR-3 cells were not significantly affected by the combined STS and DXR (herein defined as STS + DXR) treatment (Additional file 1: Fig. S1b). In line with these findings, STS also sensitized MDA-MB-231 and to a lesser extent MCF-7 cells to cisplatin, used for treatment of TNBC, at concentrations ranging from 0.5 μM to 40 μM (Additional file 1: Fig. S1d).

### Differential accumulation of reactive oxygen species in TNBC and near normal epithelial breast cells after starvation

Prompted by previous findings demonstrating that STS promotes oxidative stress in cancer cells [16], we used H_2_DCFDA to quantify ROS production in the cell models. The combination of STS with DXR resulted in an almost twofold increase of ROS production in MDA-MB-231, MDA-MB-468 and HS578. Conversely, the combined treatment did not induce ROS increase in the near normal MCF-10A cells compared to DXR-treated cells (Fig. 1c). The synergic effect of the combined treatment was specific for the TNBC cell lines since it was not observed in MCF-7 and SKBR-3 cells (Additional file 2: Fig. S2a).

Cell treatment with the ROS scavenger N-acetyl-cysteine (NAC) partially rescued MDA-MB-231 cells from the cytotoxicity associated with STS + DXR treatment (Additional file 2: Fig. S2b), indicating that ROS is a key effector of the STS-induced sensitization of the MDA-MB-231 cells to CT. To elucidate the underlying mechanism of elevated ROS production in STS + DXR treated malignant cells, we assessed the transcriptional status of the master regulator of cellular redox *NRF2* (Nuclear factor erythroid 2-related factor 2) and two of its main antioxidant downstream target genes, namely *TXNRD1* (thioredoxin reductase 1) and *NQO1* [NAD(P)H quinone dehydrogenase]. The levels of *NQO1* and *TXNRD1* were significantly decreased in DXR + STS treated TNBC cell lines in line with the elevated ROS levels observed in this setting (Additional file 2: Fig. S2c). Interestingly, in MDA-MB-231 and MDA-MB-468 cell lines, *NRF2* levels were increased, in combined STS + DXR versus DXR treatment, suggesting a compensatory expression of *NRF2* as previously described [29]. On the other hand, in the near normal context, the expression levels of *NQO1* and *TXNRD1* did not change significantly while *NRF2* mRNA levels increased between STS + DXR and DXR treatment (Additional file 2: Fig. S2c).

To confirm the role of ROS in STS-induced sensitization of cancer cells to cell death, we used TH1579 (karonudib) [30–32], a potent and highly selective MutT Human Homolog1 (MTH1) inhibitor, which was shown to selectively promote death of cancer cells by inhibition of DNA repair following oxidative stress [33]. Hence, we replaced doxorubicin with this targeted agent highly relevant in a context of oxidative stress pathway. As shown in Additional file 2: Fig. S2d, treatment with TH1579 [30–32] at 0.2 μM to 20 μM concentration enhanced STS-induced cytotoxicity in MDA-MB-231 cells. The TH1579 effect was less prominent in the survival of MCF-7 cells, whereas no significant difference was observed in the SKBR-3 cell line (Additional file 2: Fig. S2d). The enhanced cytotoxicity of the combined STS and TH1579 treatment in MDA-MB-231 cells was associated with augmented ROS production (Fig. 1d) compared to TH1579 treatment alone.

The cytotoxic effects elicited by common chemotherapeutics including doxorubicin are mainly mediated by the induction of double strand breaks (DSBs) which are the most lethal type of DNA damage. ROS are also responsible for induction of DNA damage including DSBs [34]. To test the impact of STS on the accumulation of DSBs, we stained cells with an antibody against histone H2AX phosphorylation at serine 139 (also termed γH2AX); a surrogate marker for DSBs. The number of γH2AX foci per nucleus was significantly increased in starved TNBC cell lines compared to non-starved cells (Additional file 2: Fig. S2e). STS + DXR exposure exhibited an additive effect increasing in the number of γH2AX foci in TNBC cells. Interestingly, STS partially ameliorated the DXR-induced DNA damage in MCF-10A cells, suggesting a protective effect in a non-malignant context (Additional file 2: Fig. S2e).

Collectively these data indicate that combined CT and STS treatment sensitize TNBC cells to cell death via increased ROS production associated with elevated DNA damage.

### Combined treatment decreases mitochondrial respiration while increasing the glycolytic reserve in TNBC

**Fig. 2:**
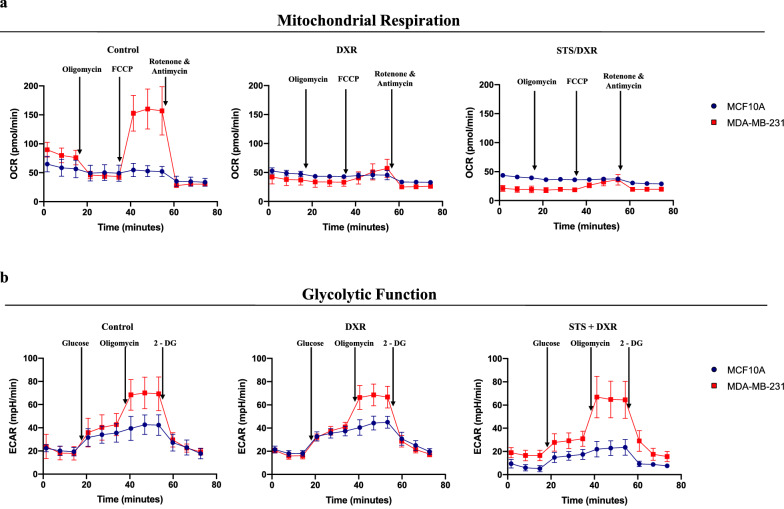
Differential metabolic responses to starvation and chemotherapy in malignant and near normal breast cell lines. **a** Oxygen consumption rate (OCR) measurements in MCF-10A and MDA-MB-231 cells treated with DXR or combined treatment (STS + DXR), using the Seahorse Analyzer. Combination treatment selectively decreases mitochondrial oxidative phosphorylation in breast cancer cells. Data are presented as mean of OCR ± SD. **b** Extracellular Acidification Rate (ECAR) measurements in MCF-10A and MDA-MB-231 cells treated with DXR or STS + DXR, using Seahorse Analyzer. Glycolytic function is preserved in DXR and is reduced in MCF-10A with STS + DXR, but not in MDA-MB-231. Data are presented as mean ECAR ± SD

Mitochondria are major source of cellular ROS production during oxidative phosphorylation (OXPHOS); the latter drives a chemical gradient that yields ATP [35]. To address whether the STS + DXR treatment compromises the mitochondrial functionality, mitochondrial respiration efficacy was evaluated. We used Seahorse cellular stress assays to measure the oxygen consumption rate (OCR) enabling a direct assessment of OXPHOS [36]. Cancer cells had a significantly higher basal OCR than MCF-10A cells (Fig. 2a). Similar findings in terms of low levels of OCR in MCF-10A have been previously described [37]. Interestingly, OCR under basal and maximal capacity (FCCP-stimulated conditions) was dramatically reduced by DXR and further minimized by STS pretreatment in MDA-MB-231 but not in MCF-10A cells (Fig. 2a). Furthermore, STS + DXR treatment decreased the spare respiratory capacity (a measure of the ability of cells to respond to elevated energy demands) and the mitochondrial coupling efficiency (the fraction of basal OCR used for ATP synthesis) in MDA-MB-231 (Fig. 2a; Additional file 3: Fig. S3a). This finding suggests that STS + DXR promotes mitochondrial respiration collapse and impairs the capacity for ATP production in the malignant context.

Seahorse assays were also used to evaluate glycolytic function by assessing the extracellular acidification rate (ECAR) [36]. Glycolytic capacity was consistently higher in MDA-MB-231 cells compared to the MCF-10A cell line (Additional file 3: Fig. S3b), supporting a greater reliance of MDA-MB-231 cells on glycolysis to meet energetic demand. STS followed by DXR treatment decreased glycolysis in MDA-MB-231, while it had little or no effect on maximal glycolytic function (Fig. 2b). Similarly, the glycolytic reserve was also significantly greater in MDA-MB-231 compared to MCF-10A cells and was further increased in malignant cells after exposure to STS + DXR (Additional file 3: Fig. S3b). The latter suggests that glycolysis may be activated in MDA-MB-231 cells to compensate for mitochondrial respiration collapse following STS. Interestingly, non-glycolytic acidification was preserved in cancer cells but was decreased in near normal cells upon combined treatment (Additional file 3: Fig. S3b), implying decreased proton production by processes other than glycolysis. Taken together, these results indicate mitochondrial dysfunctionality as a key determinate of TNBC susceptibility to STS + DXR treatment compared to near normal cells.

### Combined treatment induces differential metabolic reprogramming

Mitochondria are the site of production of biomolecules, such as nucleotides, fatty acids, cholesterol, and amino acids that are essential during hyperproliferative stress observed in cancer cells [38].

**Fig. 3:**
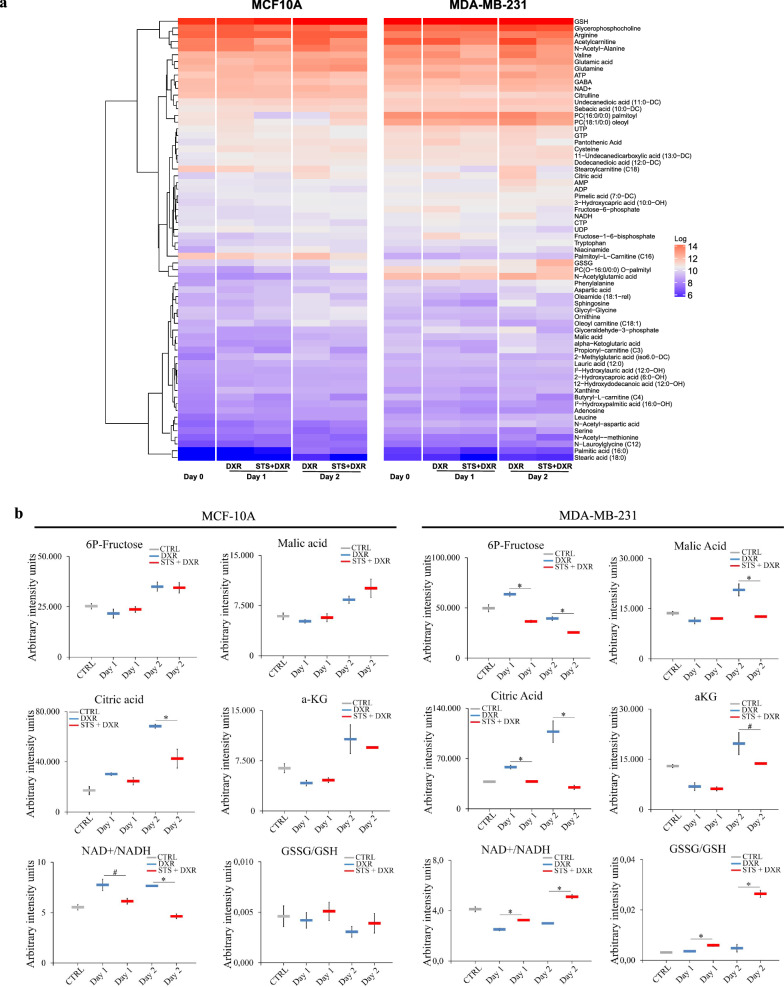

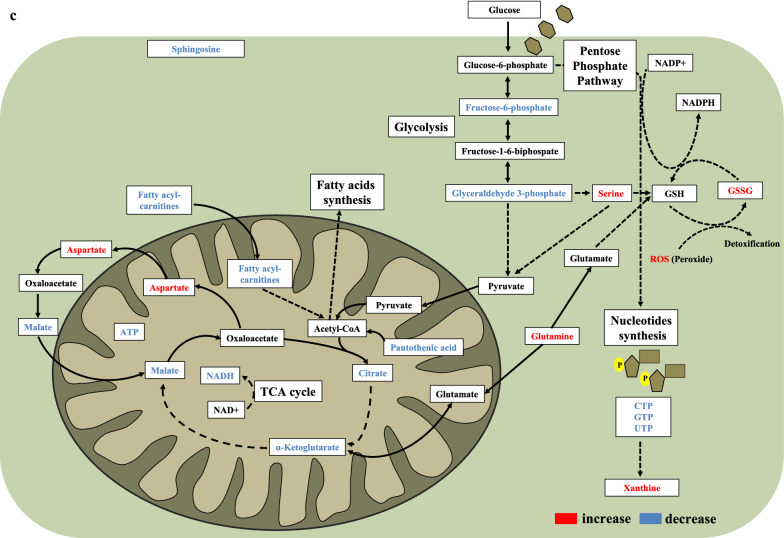
Combined starvation and doxorubicin treatment induces differential metabolic reprogramming. **a** Heatmap visualization of intracellular metabolites profile in MCF-10A and MDA-MB-231 treated with DXR or STS + DXR. Cells were harvested on day 0, day 1 and day 2 after treatment. **b** Intracellular profile of selected metabolites in MCF-10A and MDA-MB-231 treated with DXR or STS + DXR. Cells were harvested on day 0, day 1 and day 2. *P ≤ 0.05. **c** Graphical abstract depicting metabolic rewiring in triple-negative breast cancer cells upon combined STS + DXR treatment

To assess the impact of STS + DXR treatment on cell metabolism, we performed mass spectrometry metabolomics. We evaluated the levels of intracellular metabolites in MDA-MB-231 and MCF-10A cells treated with DXR or STS + DXR, harvested at 0 h (baseline), 24 h and 48 h. We quantified 66 different intracellular metabolites in both cell lines and identified significant changes in 65% (43/66 metabolites) and 56% (37/66 metabolites) between combined STS + DXR or DXR alone at 48 h in cancer and near-normal cells, respectively. MDA-MB-231 cells were characterized by a relative depletion of the tricarboxylic acid (TCA) intermediates citrate, α-ketoglutarate and malate, and the glycolysis intermediate fructose 6-phosphate upon combined treatment relative to DXR alone, an effect not observed in MCF-10A cells (Fig. 3a, b, Additional file 4: Fig. S4a). Interestingly, the α-ketoglutarate/citrate ratio was significantly increased in MDA-MB-231 cells upon the STS + DXR treatment at 48 h when compared to the DXR-treated cells (Additional file 4: Fig. S4a). In contrast, no significant differences were noted in MCF-10A cells (Additional file 4: Fig. S4a). The levels of pantothenic acid, a key precursor of Coenzyme A (CoA), were reduced in MDA-MB-231 versus MCF-10A cells upon STS + DXR (Fig. 3a, Additional file 4: Fig. S4a), which further supports the deterioration in oxidative flux from citrate to α-ketoglutarate under the combined treatment in the malignant setting. Furthermore, after 48 h of combined treatment, decreased nicotinamide adenine dinucleotide (NADH) levels were detected in MDA-MB-231 compared to MCF-10A cells (Fig. 3a, Additional file 4: Fig. S4a). This finding was associated with increased NAD + /NADH ratio in cancer cells (Fig. 3b). Consistent with the ROS analysis, the GSSG/GSH ratio was significantly elevated in cancer cells treated with the combined treatment compared to DXR alone, an effect not observed in the near normal MCF-10A cells (Fig. 3b).

Exposure of cancer cells to STS + DXR for 48 h induces increased levels of serine, decreased the concentration of the branched-chain amino acid (BCAA) valine (Fig. 3a, Additional file 4: Fig. S4a) and N-acetyl aspartic acid (also known as N-acetyl aspartate, NAA). Conversely, no notable differences were found in MCF-10A cells.

The major findings of the metabolomics analysis are summarized in Fig. 3c. In conclusion, in a model of TNBC cells, STS + DXR regimen compromises key metabolic pathways related to energy production, while it favors a metabolic switch that is associated with increased ROS production.

### Metabolic signature upon combined treatment and correlation with clinical outcome

**Fig. 4:**
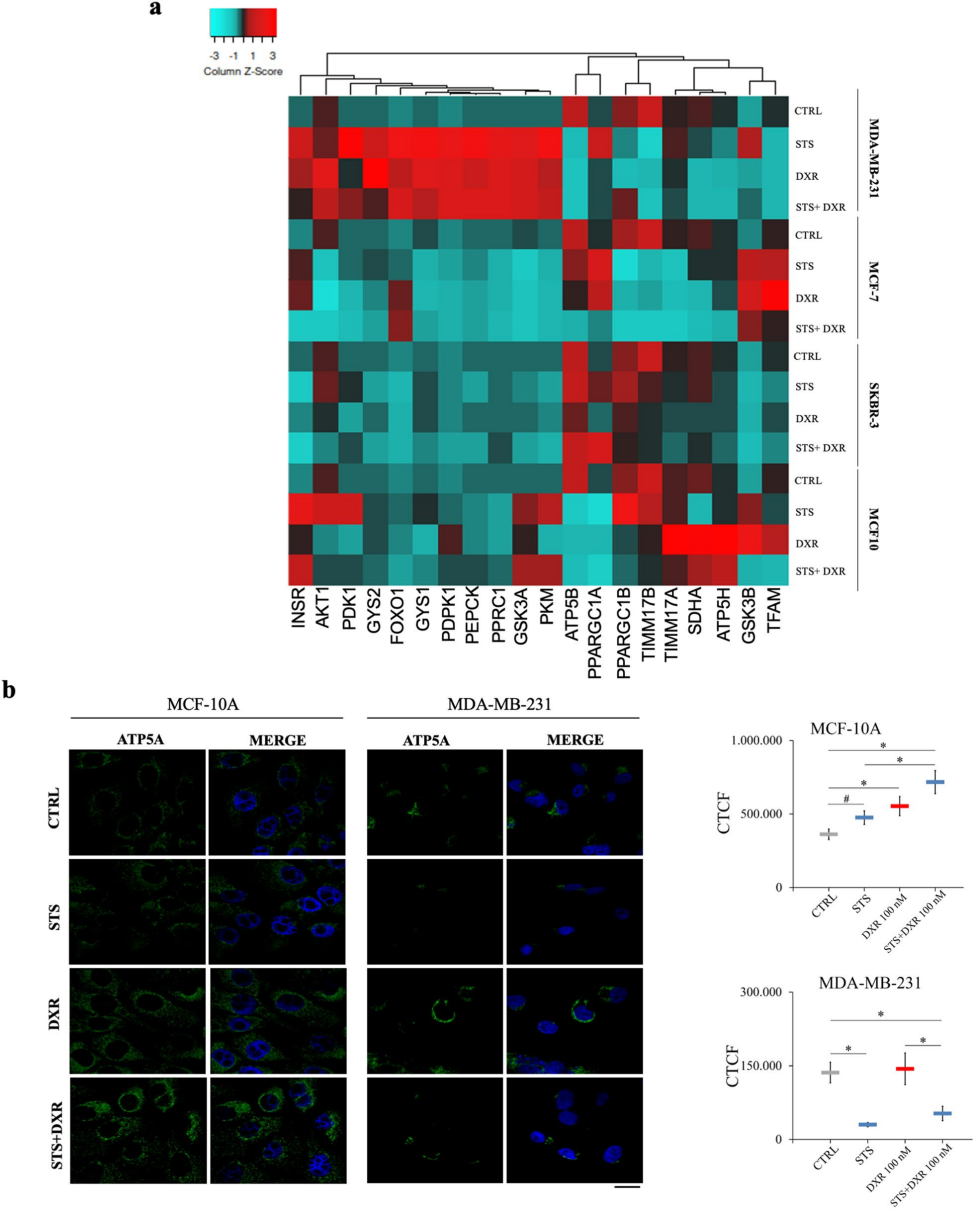

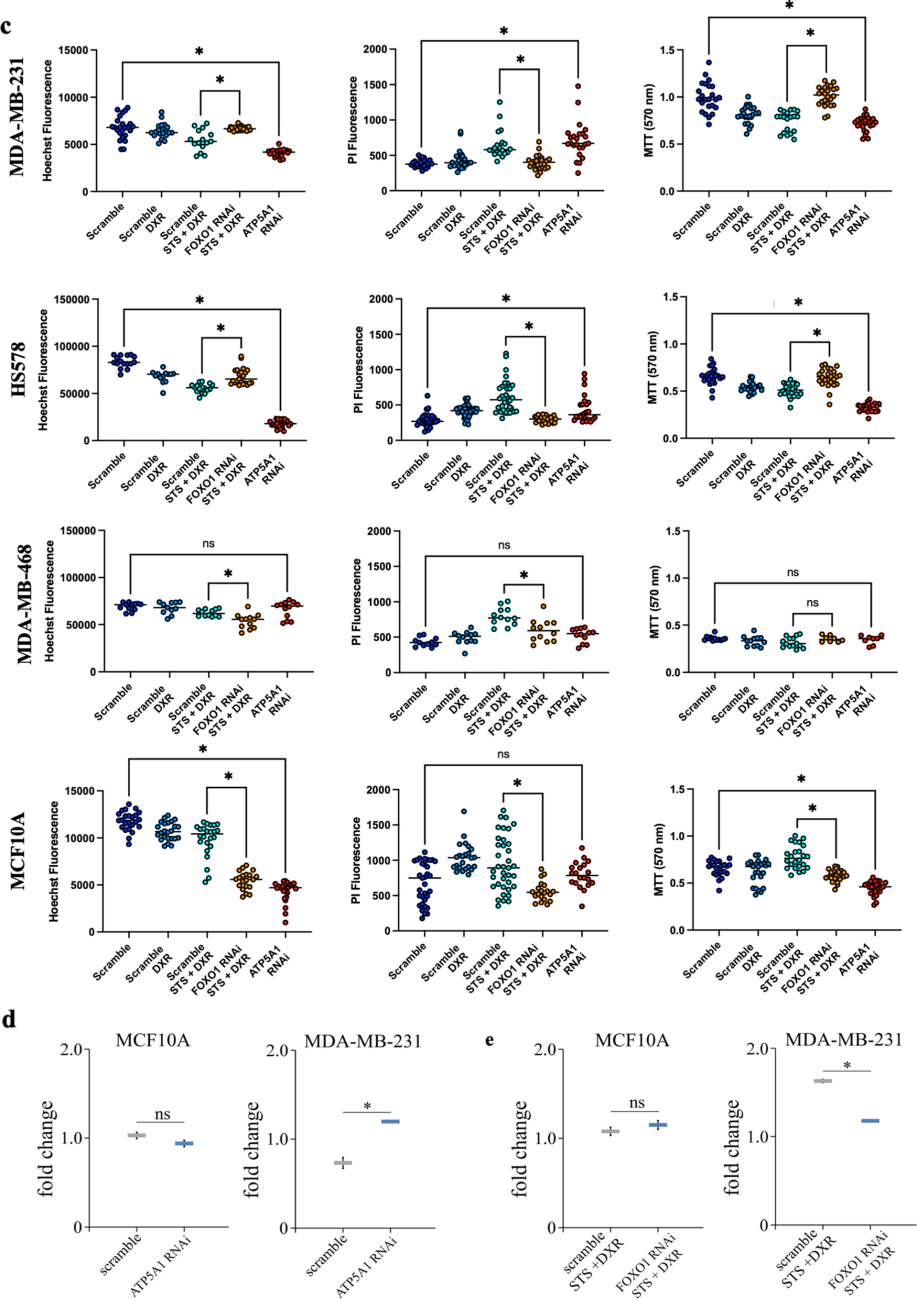
Differential functional responses to starvation and chemotherapy in malignant and near normal breast cell lines. **a** Heatmap visualization of metabolism-related gene expression in MDA-MB-231, MCF-7, SKBR-3 and MCF-10A cells. Samples are arranged by treatment group. Each sample was measured in duplicate for each gene. Expression signature appears to be cell-specific and differentially affected by treatments.** b** Expression of ATP5A in breast epithelial cell lines upon STS, DXR or STS + DXR assessed by immunofluorescence analysis. Left panel: representative micrograph where ATP5A is shown in green, nuclei were counterstaining with DAPI (blue). Scale bar, 20 μm. Right panel: Quantification, where data are presented as mean corrected total cell fluorescence (CTCF) ± SD. *P ≤ 0.05. **c** *ATP5A1* silencing mimicked the effect of STS + DXR treatment resulting in reduced cell viability in MDA-MB-231 and HS578, as demonstrated by lower Hoechst, higher PI and lower MTT signal in comparison to Scramble. *FOXO1* silencing eliminate the selective sensitivity of combined STS + DXR treatment demonstrated by elevated cellular viability in MDA-MB-231 and HS578 cells as shown by higher Hoechst, lower PI and higher MTT signal in comparison to ‘Scramble RNAi STS + DXR’. **d** Assessment of intracellular ROS production in MCF-10A and MDA-MB-231 cells with or without *ATP5A1* silencing. Data are presented as mean of the fold change ± SD. *P ≤ 0.05. **e** Assessment of intracellular ROS production in STS + DXR treated MCF-10A and MDA-MB-231 cells with or without *FOXO1* silencing. Data are presented as mean of the fold change ± SD. *P ≤ 0.05

To further validate the observed differences in metabolic responses, we examined the transcriptional levels of 21 genes involved in glucose metabolism and mitochondrial function. In contrast to near normal epithelial cells, cancer cells were shown to down-regulate most mitochondrial genes upon STS + DXR exposure in a cell-specific manner. The most substantial decrease in MDA-MB-231 and/or MCF-7 cells upon STS + DXR treatment compared to MCF-10A was noted for the mitochondrial transcription factor A (*TFAM*) (a key activator of mitochondrial transcription and mitochondrial DNA replication regulator), *SDHA* (encoding succinate dehydrogenase flavoprotein subunit A), as well as *ATP5H* and *ATP5B* (two genes encoding the ATP synthase subunits) (Fig. 4a). Immunofluorescence analysis further confirmed that STS alone, or in combination with DXR, resulted in decreased levels of the ATP synthase lipid-binding protein subunit alpha (ATP5A), which encodes the catalytic core of F_1_F_0_-ATPase, in MDA-MB-231, MDA-MB-468, HS578 and MCF-7 cells, while no significant differences were observed in SKBR-3 cells (Fig. 4b; Additional file 4: Fig. S4b, c). Conversely, STS + DXR exposure led to increased ATP5A staining in MCF-10A cells highlighting the protective role of STS on mitochondrial function. Along this line, *ATP5A1* (also known as *ATP5A*) silencing (Additional file 5: Fig. S5a) recapitulated the effect of combined STS and CT treatment in MDA-MB-231 and HS578 cells as demonstrated by increased cytotoxicity (Fig. 4c,), elevated ROS levels (Fig. 4d) and increased γH2AX foci (Additional file 5: Fig. S5c). *ATP5A1* silencing also recapitulated with the decreased mRNA levels of *TXNRD1*, while *NRF2* was elevated suggesting a compensatory response in MDA-MB-231 cells as previously described [29] (Additional file 5: Fig. S5e). Similarly, *ATP5A1* silencing resulted in decreased cell viability of MCF10A. Conversely MDA-MB-468 cells show very little response to siRNA treatment (Fig. 4c).

Collectively, these data fully support the STS-dependent compromised mitochondrial respiration in TNBC cells (Fig. 2a).

The STS + DXR treatment promoted increased expression of genes associated with adaptation to nutrient deprivation (e.g. phosphoenolpyruvate carboxykinase, *PEPCK*, regulating gluconeogenesis*,* in MDA-MB-231 cells) or oxidative stress (e. g. Forkhead box protein O1, *FOXO1,* in MCF-7 cells) and *PPARGC1A* (also known as PGC-1α, the master regulator of mitochondrial biogenesis and also required for the induction of antioxidant responses [39] in SKBR-3) (Fig. 4a).

**Fig. 5:**
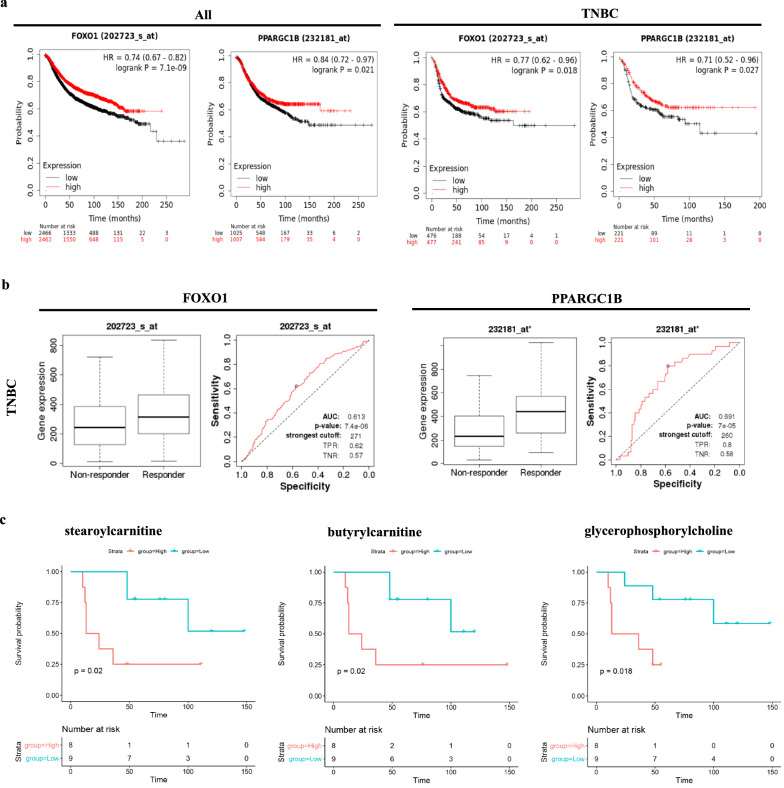
Metabolic signature upon starvation with doxorubicin and correlation with clinical outcome. **a** Kaplan–Meier survival curves depicting that elevated levels of *FOXO1* and *PPARGC1B* are associated with prolonged relapse-free survival in all breast cancer breast subtypes (All) including TNBC. **b** ROC curve analysis depicting that elevated levels of *FOXO1* and *PPARGC1B* are associated with improved response to anthracycline treatment in TNBC. **c** Kaplan–Meier survival plots depicting the correlation between intracellular concentrations of the selected metabolites with patient’s prognosis in a TNBC cohort. Decreased levels of stearoylcarnitine, butyrylcarnitine and glycerophosphorylcholine being observed upon STS + DXR in MDA-MB-231 cells, are all associated with favorable clinical outcome

We next examined the prognostic relevance of these 21 genes by integrating transcriptomic data from the TCGA repository and employing Kaplan–Meier plotter [24]. Notably, from the 21 genes tested, we identified higher expression of *FOXO1* and *PPARGC1B* (Peroxisome Proliferator-Activated Receptor Gamma, Coactivator 1 Beta, alternatively known as PGC-1β) as independent markers related with relapse-free survival (RFS) in breast cancer patients irrespective of breast cancer subtypes, including TNBC (Fig. 5a). Interestingly, employing ROC plotter [26], we found that increased expression of *FOXO1* and *PPARGC1B* were validated as predictive markers associated with improved response to anthracycline treatment in TNBC (Fig. 5b). To validate these findings in the cell line models, *FOXO1* was silenced by iRNA (Additional file 5: Fig. S5b), and this eliminated the selective sensitivity of the combined STS + CT treatment. Specifically, *FOXO1* silencing reduced cytotoxicity to combined STS + DXR treatment in MDA-MB-231 and HS578 cell lines, (Fig. 4c), that was associated with reduced ROS levels (Fig. 4e) and decreased DNA damage depicted by reduced γH2AX foci (Additional file 5: Fig. S5d). Accordingly, *FOXO1* silencing was associated with elevated levels of *NQO1* and *TXNRD1* in STS + DXR treated MDA-MB-231 cells (Additional file 5: Fig. S5f). On the other hand, MDA-MB-468 cell lines responded differently to siRNA treatment which could be dependent on different metabolic needs of each cell line (Fig. 4c).

We also observed that decreased levels of stearoylcarnitine, butyrylcarnitine and glycerophosphorylcholine are associated with better overall survival (Fig. 5c) in 17 patients of the TNBC cohort [40], mirroring the decreased levels of these metabolites observed in MDA-MB-231 cells subjected to STS + DXR (Additional file 4: Fig S4a).

These data highlight the dependency of cancer cells on nutrient and oxidative stress adaptation in patients, which is severely compromised by the STS + DXR treatment, supporting the relevance of the in vitro metabolic and transcriptional analysis.

### Intermittent caloric restriction combined with chemotherapy retards tumor growth and suppresses metastatic potential in a TNBC mouse model

To further validate the in vitro findings, we assessed the effect of short-term caloric restriction on a TNBC syngeneic orthotopic mouse model. We used the highly metastatic mouse 4T1 cell line [41], which corresponds to the human MDA-MB-231 cell line.

**Fig. 6:**
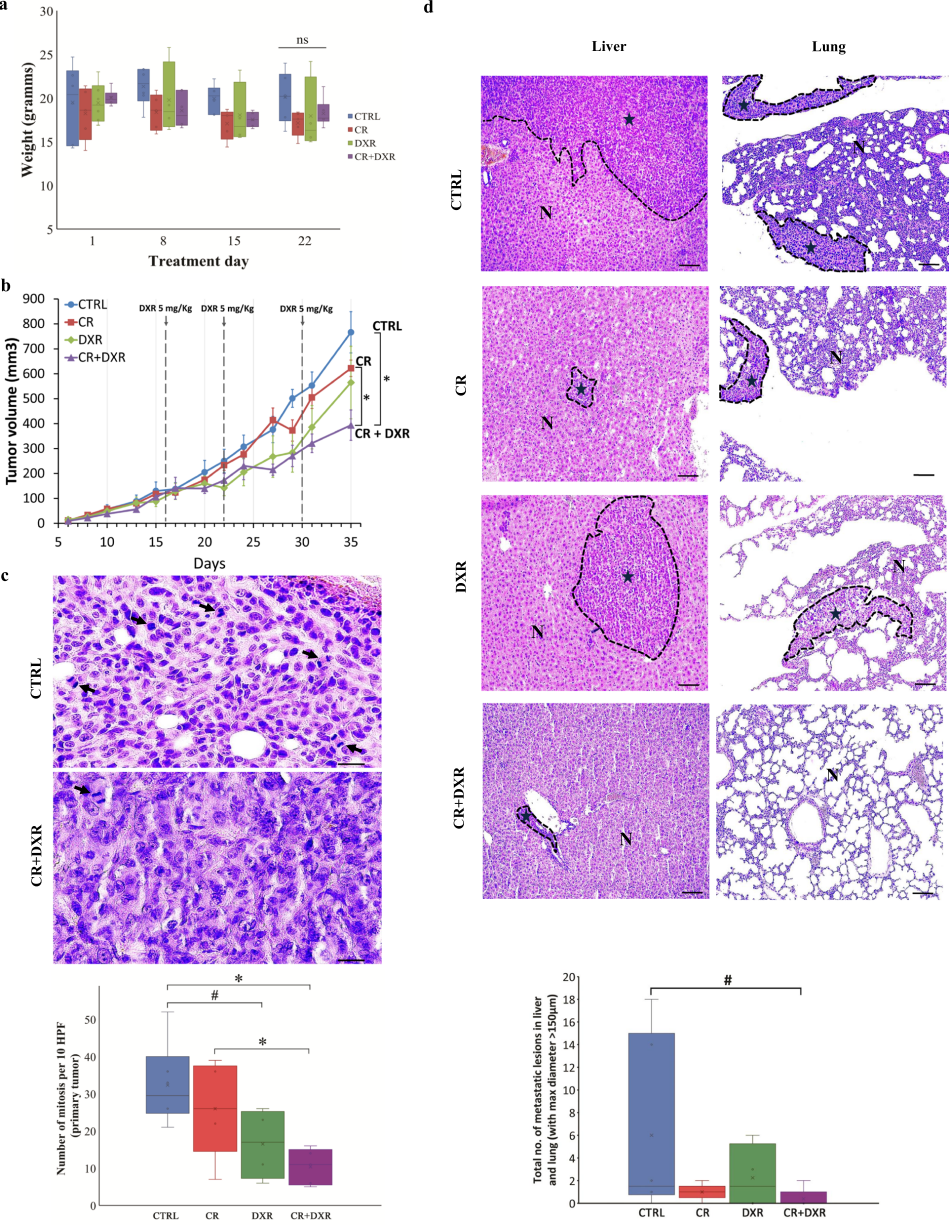
Caloric restriction combined with chemotherapy retards tumor growth and suppresses metastatic potential in a triple-negative breast cancer mouse model. **a** Graph demonstrating average animal weight per treatment group in four timepoints during experiment. No significant changes were observed in either treatment group. Data are presented as mean ± SD. ns: non-significant. **b** Tumor-bearing mice were untreated (CTRL) or treated with caloric restriction 30% (CR), doxorubicin 5 mg/kg weekly (DXR) or combination (CR + DXR). Tumor growth curves for each condition are shown. Data are presented as mean ± SD. *P ≤ 0.05. **c** Upper panel: Representative high-power field (HPF, magnification 400x) micrograph of hematoxylin & eosin (H&E) staining tissue. Arrows indicate mitotic cells in the untreated CTRL and combined CT + DXR group. Scale bar, 20 μm. Bottom panel: quantification of the number of mitotic cells per 10 HPF in H&E sections of the primary tumor in all four subgroups. Data are presented as mean + SD. *P ≤ 0.05; #statistical trend. **d** Representative microscopy H&E staining images from liver and lung in all four subgroups. Black stars denote metastatic foci enclosed with dashed lines. N: normal parenchyma. Scale bar, 100 μm. Bottom panel: quantification of the total number of metastatic lesions in liver and lung with max. diameter > 150 μm examined in hematoxylin & eosin (H&E) sections among the four subgroups. Combination treatment reduces metastatic tumor burden in 4T1 syngeneic model. Data are presented as mean ± SD. #statistical trend

STS sensitized 4T1 cells to DXR and further increased the levels of DXR-induced ROS (Additional file 6: Fig. S6a, b). We injected 4T1 cells into syngeneic BALB/c mammary fat pads. To overcome possible side effects of complete fasting, we subjected mice to a hypocaloric diet in the form of 30% caloric restriction (CR) alone, DXR treatment, or combined treatment for three consecutive cycles. Control group mice were fed ad libitum throughout the 35-day cycle and reached a maximum weight of 20.1 ± 3.9 g. Mice subjected to cycles of DXR, CR and combined CR + DXR treatment lost between 5 and 19% of their initial body weight by the end of the third cycle (Fig. 6a; Additional file 7: Table S1). The lack of significant weight loss in these mice could be attributed to the hypocaloric diet regimen, which is milder than STS, and to the fact that mice were fed ad libitum between the therapeutic cycles. No mice were sacrificed before the experimental endpoint.

Biochemical analysis of key markers reflecting potential systemic imbalances including LDH, Creatinine, Urea, AST (ASAT), ALT (ALAT), Potassium, CPK as well as the malnutrition marker albumin, did not reveal significant differences between the different groups suggesting that the dietary approach was well-tolerated (Additional file 6: Fig. S6c). As expected, the levels of serum glucose were decreased upon CR and CR + DXR treatment as previously reported (Additional file 6: Fig. S6c) [42].

Injection of 4T1 cells in BALB/c resulted in the development of tumors in fat pads and the development of advanced metastatic disease in eleven mice. At the endpoint, primary tumor growth was inhibited by both CR and DXR alone to a similar degree and this effect was maximized by the CR + DXR regimen (Fig. 6b; Additional file 6: Fig. S6d). Notably, CR combined with DXR decreased the mitotic index (Fig. 6c), an independent universal prognostic factor in human breast cancer [43]. Further, CR + DXR restrained metastatic potential in two distant organs (lung and liver) (Fig. 6d). Finally, using oxygraphy, we investigated the effect of CR + DXR treatment on the metabolic status of tumors in our mouse model and found a decline in respiration state 3 (indication of the rate of ATP consumption) and respiration state 4 (indication of the coupling effect) (Additional file 6: Fig. S6e). Altogether, these findings demonstrate the safety and efficacy of cycles of combined hypocaloric diet and CT to treat TNBC tumors at an in vivo setting.

## Discussion

The use of chemotherapy treatment is often limited by toxic side-effects caused to healthy cells, limiting their usefulness [44]. However, an enormous amount of research and development are necessary for the approval of new anticancer drugs, and even upon entering into clinical practice, efficacy may still be limited to a relatively small patient subgroup [45]. Therefore, a crucial step in the improvement of cancer treatment is to assess whether approved drugs can be integrated into new therapeutic regimens that maximize their efficacy while reducing toxicity on healthy tissues. In this context, our data provide a detailed metabolic analysis to assess how STS potentiate the cytotoxic effect of chemotherapeutic agents that causes DNA damage (DXR or CIS) or inhibit oxidative stress-induced DNA damage repair (MTH1 inhibitor) in TNBC cells, while protecting non transformed mammary cells in vitro (Fig. 1, Additional file 1: Fig. S1a, Additional file 2: Fig. S2). Our data point to mitochondrial disfunction as the main STS targeted process. The synergist effect of CR + DXR was further proven in an in vivo orthotopic mouse model of TNBC in a regimen of CT, which was well-tolerated (Fig. 6).

One of the most important findings of this study is the protective effect of STS on near normal non-transformed MCF-10A cells. STS + DXR treatment did not increase oxidative stress and DNA damage in this cell line (Fig. 1, Additional file 2: Fig. S2). Our findings are in agreement with previous studies demonstrating that STS promotes ROS production in cancer cells and protects normal cells by decreasing CT-induced DNA damage [10, 16, 46, 47]. However, we have expanded these observations and demonstrated that the differential response is associated with metabolic rewiring, altered levels of metabolites, differential oxidative phosphorylation and altered redox status (Figs. 1, 2, 3, 4).

Expression of key genes involved in mitochondrial biogenesis and activity, such as *ATP5A*, *ATP5B, PGC-1α, TFAM*, and *SDHA* are downregulated upon STS + DXR treatment in TNBC cells (Fig. 4; Additional file 4: Fig. S4). Interestingly, silencing of *ATP5A* recapitulated the effect of STS + DXR treatment in MDA-MB-231 and HS578 TNBC cell lines, supporting that mitochondrial dysfunctionality is a key determinate of TNBC susceptibility to combined STS and chemotherapy treatment (Fig. 4; Additional file 5: Fig. S5). Up-regulation of *PGC-1α* expression was shown to enhance OXPHOS, elevate ATP production and increase invasiveness in 4T1 breast cancer cells, and it is associated with increased formation of distant metastasis in breast cancer patients [48]. Thus, STS leads to reduced *PGC-1α* expression*,* and correlates with the reduced metastatic capacity in mice treated with the CR + DXR treatment, presented here (Figs. 4, 6).

A previous study in the CT26 colon carcinoma cell line has demonstrated that STS combined with CT led to reduced glycolysis and increased OCR, indicating elevated OXPHOS. However, overall ATP levels were reduced due to elevated ROS production [49]. In contrast we demonstrated in MDA-MB-231 breast cancer cells that glycolytic reserve was increased, suggesting that glycolysis compensate for the collapse in OXPHOS. This difference may be attributed to the different cancer cell lines and/or tissue origin of the tumor examined. Hence, it is important to use our approach to interrogate the metabolic consequences of STS in other cancer cell models.

The elevated levels of ROS observed upon STS + DXR treatment are directly associated with the elevated GSSG/GSH and NAD + /NADH ratio in cancer cells, while this effect was not observed in MCF-10A cells. The increased NAD + /NADH ratio was mainly attributed to down-regulation of NADH, which can be due to the reduced glycolytic intermediates in cancer cells (Fig. 3) [50]. NADH may also be consumed to regenerate GSH to compensate for the STS-dependent increased ROS production in MDA-MB-231 cells (Fig. 3). In this scenario, the increased level of serine may reflect a feedback mechanism that contributes to NADPH production, which in turn is used for maintaining GSH in the reduced state [51]. In addition, given that serine is a single carbon donor that promotes cysteine synthesis, which is a precursor of GSH [7], increased serine levels may further support the hyperactivation of antioxidant response in TNBC cells upon DXR-STS treatment. An additional source of ROS production in MDA-MB-231 cells upon combined treatment could be attributed to increased activity of xanthine oxidase (XO) [52], suggested by the higher production of xanthine (Fig. 3).

We can conclude that, in MDA-MB-231 cells, STS imposes mitochondrial metabolic rewiring that aims, to buffer unsuccessfully the increase of ROS (Fig. 3).

The synergistic effect of STS on enhanced ROS production was observed in TNBC cell lines including MDA-MB-231, MDA-MB-468 and HS578 cell lines, and to a limited extend in the luminal MCF-7 cells. From a mechanistic perspective combined STS + DXR treatment in TNBC cell lines was associated with decreased mRNA levels of *NQO1* and *TXNRD1* which are downstream transcriptional targets of NRF2 (Additional file 2: Fig. S2), coming in line with the elevated ROS production. In contrast, ROS production was not affected in SKBR-3, the HER2 overexpressing cell line (Additional file 2: Fig. S2). The involvement of HER2 overexpression in attenuating oxidative stress in breast cancer suggests that HER2 signaling regulates oxidative balance [53]. This is further supported by observed increase of *PGC-1α* expression in SKBR-3 cells exposed to STS + DXR, which could help to compensate the oxidative challenge posed by an STS-induced ROS increase (Fig. 4).

MDA-MB-231 cells were characterized by a relative depletion of the tricarboxylic acid (TCA) intermediates citrate, α-ketoglutarate and malate upon combined treatment relative to DXR alone, an effect not observed in MCF-10A cells (Fig. 3a–c; Additional file 4: Fig S4). In principle Krebs intermediates can be lost to catapletoric pathways or replaced by anaplerotic pathways. Specifically, α-ketoglutarate is required for glutamine biosynthesis which is a precursor for GSH a key enzyme for anti-oxidant defense. The increased glutamine levels associated with the elevated GSSG/GSH ratio in combined STS + DXR versus only DXR treated MDA-MB-231 cells (Fig. 3a–c; Additional file 4: Fig. S4), suggests that α-ketoglutarate depletion could be attributed to an increased need for glutamine and GSH biosynthesis due to elevated ROS in MDA-MB-231 cells after STS + DXR treatment. Citric acid is a precursor for lipid biosynthesis. It is well-established that cancer cells harness lipid metabolism [54], providing an explanation for the decreased citric acid levels in combined STS + DXR context. Furthermore, as citric acid is a metabolic precursor of α-ketoglutarate, depletion of citric acid could be explained due to the increased demand for α-ketoglutarate production. Alternatively, and not mutually exclusive, the decreased levels of citric acid could provide an additional explanation for the partial depletion of α-ketoglutarate. Malate is a part of malate-aspartate shuttle. Notably, aspartate, has a critical role for proliferation of cancer cells [55]. In combined STS + DXR context, MDA-MB-231 cells had increased aspartate levels (Fig. 3a, c), providing an explanation for the decreased malate levels.

From a clinical perspective combined STS + DXR resulted in elevated *FOXO1* and *PPARGC1B* (*PGC-1β*) levels, which were found to be independent factors of prolonged Relapse Free Survival (RFS) and improved response to anthracyclines in the TNBC cohort (Fig. 5). Along this line *FOXO1* silencing eliminate the selective sensitivity of combined STS + DXR treatment in MDA-MB-231 and HS578 TNBC cell lines (Fig. 4; Additional file 5: Fig S5). These findings substantiate from a cellular and molecular perspective the prognostic and predictive impact of FOXO1 in triple negative breast cancer patients (Fig. 5). FOXO1 regulates metabolic homeostasis, upon response to STS and oxidative stress [56], induces hepatic gluconeogenesis and promotes autophagy in response to oxidative stress [57]. In agreement with this, we found that STS + DXR induced an autophagic response in MDA-MB-231 cells but not in near normal MCF-10A cells (data not shown). A key function of PPARGC1B is to control mitochondrial biogenesis [58] and it promotes OXPHOS along with fat oxidation, suggesting a compensatory strategy for compromised mitochondrial respiration.

Collectively, our findings show that STS induces oxidative and metabolic stress in cancer cells. Along the same lines, genotoxic stress promotes OXPHOS as an adaptive mechanism to support energy demands for DNA repair and to restore energy homeostasis [59]. Given that cancer cells are known to be under DNA damage stress, STS becomes a very efficient approach to deplete energy stores rendering them vulnerable to genotoxic agents.

## Conclusions

To understand cancer-specific vulnerability, we performed a comprehensive analysis combining nutrient and caloric deprivation with conventional chemotherapeutic drug treatments in TNBC and near normal settings in vitro and in vivo. We found that STS increased CT-induced cytotoxicity in TNBC cell lines through ROS up-regulation, while protecting near normal breast cells. These novel mechanistic findings provide opportunities for innovative translational applications. The synergistic effect of STS with lower CT doses offers a great advantage, since it could reduce the CT side effects. Understanding the molecular mechanisms that underline the differential response of normal versus cancer cells to dietary restriction may help to overcome CT resistance of various types of cancer, leading to safe and effective anti-cancer treatments.

Our data are in line with two studies demonstrating the beneficial role of nutrient depletion in sensitizing cancer cells to estrogen therapy and in depleting TNBC stem cells, highlighting the efficiency of caloric restriction in cancer treatment that extends beyond chemotherapy [60, 61]. Together with the results from the first randomized controlled study proving the enhanced therapeutic efficiency of fasting mimicking diet in patients with HER2-negative breast carcinoma [13], our data supports the necessity of robust clinical trials to validate the therapeutic benefit of the combined approach and establish dietary recommendations as an adjunct to chemotherapy for triple negative breast cancer treatment.

## Supplementary Information

**Additional file 1: Fig. S1.** Starvation preferentially sensitizes different breast cancer subtypes versus near normal cells to chemotherapy. a. In vitro treatment overview: cells were glucose/serum starved (STS) for 48 h (blue bar), drugs were added during the last 24h (red bar). b. MTT survival assays after 48 h treatment with STS with or without addition of doxorubicin (DXR) in near normal MCF-10A cells and the indicated breast cancer cell lines. Data are presented as mean survival percentage. *P ≤ 0.05. c. MTT survival assay after 48 h treatment with STS with or without 100 nM doxorubicin (DXR) in near normal MCF10A cells and the triple negative breast cancer cells MDA-MB-231, MDA-MB-468 and HS578. Data are presented as mean survival percentage. *P ≤ 0.05. d. MTT survival assays after 48 h treatment with STS with or without addition of cisplatin (CIS) in near normal MCF-10A cells and the indicated breast cancer cell lines. Data are presented as mean survival percentage. *P ≤ 0.05.

**Additional file 2: Fig. S2.** Differential accumulation of reactive oxygen species in malignant and near normal epithelial breast cells after starvation. a. Assessment of intracellular ROS production; fold change of intracellular ROS production compared to control in breast cancer cell lines upon STS, DXR or combined treatment (STS+DXR). Data are presented as mean fluorescent intensity +SD. *P ≤ 0.05. b. MTT survival assay after 48 h treatment with combined STS+DXR in absence or presence of NAC (4mM) in MDA-MB-231 cells. Data are presented as mean survival percentage. *P ≤ 0.05. c. Transcriptional analysis of *NRF2* coupled with the downstream transcriptional targets *NQO1* and *TXNRD1* in the presented conditions in MCF-10A, MDA-MB-231, MDA-MB-468 and HS578 cell lines. Data are presented as mean expression values. *P ≤ 0.05. d. MTT survival assays after 48 h treatment with STS with or without addition of ΤΗ1579 in near normal MCF-10A cells and the indicated breast cancer cell lines. Data are presented as mean survival percentage. *P ≤ 0.05. e. γH2AΧ immunofluorescence staining in MCF-10A, MDA-MB-231, MDA-MB-468 and HS578 in Control (CTRL), DXR, STS and STS+DXR. Nuclei counterstained with DAPI. Scale bar, 5μm. Data are presented as mean of the number of foci per cell. *P ≤ 0.05.

**Additional file 3: Fig. S3.** Combined treatment decreases oxidative phosphorylation and increases glycolytic reserve in highly aggressive TNBC cells. a. Oxygen Consumption Rate (OCR) measurements in MCF-10A and MDA-MB-231 cells treated with DXR alone or in combination with STS, using Seahorse Analyzer. Combination treatment selectively reduces basal respiration (corresponding to basal OCR), maximal respiration (corresponding to FCCP response) and spare respiratory capacity (the difference between maximal respiration and basal respiration). b. Extracellular Acidification Rate (ECAR) measurements in MCF-10A and MDA-MB-231 cells treated with DXR alone or in combination with STS, using Seahorse Analyzer. Combination treatment selectively increases glycolytic capacity, glycolytic reserve and non-glycolytic acidification in breast cancer cells. Data are presented as mean ECAR ±SD. Ns: non-significant; *P ≤ 0.05.

**Additional file 4: Fig. S4.** Combined treatment induces differential metabolic reprogramming. a. Intracellular metabolites profile in MCF-10A and MDA-MB-231 treated with DXR or STS+DXR. Cells were harvested on day 0, day 1 and day 2. *P ≤ 0.05. b. Expression of ATP5A in triple negative cell lines MDA-MB-468 and HS578 upon DXR or STS+DXR assessed by immunofluorescence analysis. Left panel: representative micrograph where ATP5A is shown in green, nuclei were counterstained with DAPI (blue). Scale bar, 20 μm. Right panel: Quantification, where data are presented as mean corrected total cell fluorescence (CTCF) ±SD. *P ≤ 0.05. c. Expression of ATP5A in MCF-7 and SKBR-3 cell lines upon DXR or STS+DXR assessed by immunofluorescence analysis. Left panel: representative micrograph where ATP5A is shown in green, nuclei were counterstained with DAPI (blue). Scale bar, 20 μm. Right panel: Quantification, where data are presented as mean corrected total cell fluorescence (CTCF) ±SD. *P ≤ 0.05. #Statistical trend.

**Additional file 5: Fig. S5.** *ATP5A1 *silencing mimicks the effect of combined STS+DXR treatment in TNBC cells - *FOXO1* silencing eliminate the selective sensitivity of TNBC cells to STS+DXR treatment. a. Transcriptional levels of *ATP5A1* upon *ATP5A1 *silencing in MCF10A and MDA-MB-231. Data are presented as mean expression values. *P ≤ 0.05. b. Transcriptional levels of *FOXO1* upon *FOXO1* silencing in MCF10A and MDA-MB-231. Data are presented as mean expression values. *P ≤ 0.05. c. γH2AΧ immunofluorescence staining foci per cell in MCF-10A and MDA-MB-231 cells with or without *ATP5A1* silencing. Nuclei counterstained with DAPI. Scale bar, 5μm. Data are presented as mean of the number of foci per cell. *P ≤ 0.05. d. γH2AΧ immunofluorescence staining foci per cell in STS+DXR treated MCF-10A and MDA-MB-231 cells with or without *FOXO1* silencing. Nuclei counterstained with DAPI. Scale bar, 5μm. Data are presented as mean of the number of foci per cell. *P ≤ 0.05. e. Transcriptional analysis of *NRF2* coupled with the downstream targets *NQO1* and *TXNRD1* in MCF-10A and MDA-MB-231 cell lines with or without *ATP5A1* silencing. Data are presented as mean expression values. *P ≤ 0.05. f. Transcriptional analysis of *NRF2* coupled with the downstream targets *NQO1* and *TXNRD1* in STS+DXR treated MCF-10A and MDA-MB-231 cell lines with or without *FOXO1* silencing. Data are presented as mean expression values. *P ≤ 0.05.

**Additional file 6: Fig. S6.** Caloric restriction combined with chemotherapy retards tumor growth and suppresses metastatic potential in a triple-negative breast cancer mouse model. a. MTT survival assays of 4T1 mouse breast cancer cells treated with DXR alone or in combination with STS (STS+DXR). Data are presented as mean survival percentage. *P ≤ 0.05. b. Assessment of intracellular ROS production. Data are presented as mean of the fold change ±SD of intracellular ROS production in cells upon STS, DXR or combined STS+DXR. *P ≤ 0.05. c. Levels of key biochemical markers measured in serum of mice injected with 4T1 cells and treated with CR, DXR or combined CR+DXR treatment. Markers were measured in three mice from each treatment group. Data are presented as mean ±SD. One Way Anova-Kruskal Wallis test. d. Primary whole-tumor pathological specimen collected from control mice and mice treated with CR+/-DXR and euthanized at day 35. e. Mitochondria from tumor derived from control mice and mice treated with CR+/-DXR were isolated and evaluated with oxygraphy for respiration parameters. Data are presented as mean ±SD. #P<0.01 (statistical trend); *P ≤ 0.05.

**Additional file 7: Table. S1.** Weight (in grams) monitoring in control, caloric restricted (CR), DXR treated and CR+DXR treated mice.

**Additional file 8.** Methods.

## Data Availability

Raw data are available upon reasonable request.

## Abbreviations

ATP5A: ATP Synthase F1 Subunit Alpha
ATP5H: ATP Synthase Peripheral Stalk Subunit D
ATP: Synthase lipid-binding protein subunit alpha
BCAA: Branched-chain amino acid
CIS: Cisplatin
CoA: Coenzyme A
CR: Caloric restriction
CT: Chemotherapy
DSR: Differential stress resistance
DXR: Doxorubicin
ECAR: Extracellular acidification rate
FOXO1: Forkhead box protein O1
H_2_DCFDA: 2',7'-Dichlorodihydrofluorescein diacetate
MTH1: MutT Human Homolog1
NAA: N- acetyl aspartate
NAC: N-acetyl-cysteine
NQO1: NAD(P)H quinone dehydrogenase
NRF2: Nuclear factor erythroid 2-related factor
OCR: Oxygen consumption rate
OXPHOS: Oxidative phosphorylation
PEPCK: Phosphoenolpyruvate carboxykinase
PI: Propidium Iodide
PPARGC1B: Peroxisome Proliferator-Activated Receptor Gamma, Coactivator 1 Beta
RFS: Relapse Free Survival
ROS: Reactive oxygen species
RT: Room temperature
STS: Starvation
SDHA: Succinate dehydrogenase complex flavoprotein subunit A
TFAM: Mitochondrial transcription factor A
TNBC: Triple negative breast cancer
TXNRD1: Thioredoxin reductase 1
