# Supplementary material for: Short term starvation potentiates the efficacy of chemotherapy in triple negative breast cancer via metabolic reprogramming
Source: J Transl Med. 2023 Mar 3;21:169. doi: 10.1186/s12967-023-03935-9 (PMC9983166; doi:10.1186/s12967-023-03935-9)
Supplement: Supplementary file 1 — Additional file 1: Fig. S1. Starvation preferentially sensitizes different breast cancer subtypes versus near normal cells to chemotherapy. a. In vitro treatment overview: cells were glucose/serum starved (STS) for 48 h (blue bar), drugs were added during the last 24h (red bar). b. MTT survival assays after 48 h treatment with STS with or without addition of doxorubicin (DXR) in near normal MCF-10A cells and the indicated breast cancer cell lines. Data are presented as mean survival percentage. *P ≤ 0.05. c. MTT survival assay after 48 h treatment with STS with or without 100 nM doxorubicin (DXR) in near normal MCF10A cells and the triple negative breast cancer cells MDA-MB-231, MDA-MB-468 and HS578. Data are presented as mean survival percentage. *P ≤ 0.05. d. MTT survival assays after 48 h treatment with STS with or without addition of cisplatin (CIS) in near normal MCF-10A cells and the indicated breast cancer cell lines. Data are presented as mean survival percentage. *P ≤ 0.05. [file 12967_2023_3935_MOESM1_ESM.ppt]

## Slide 1
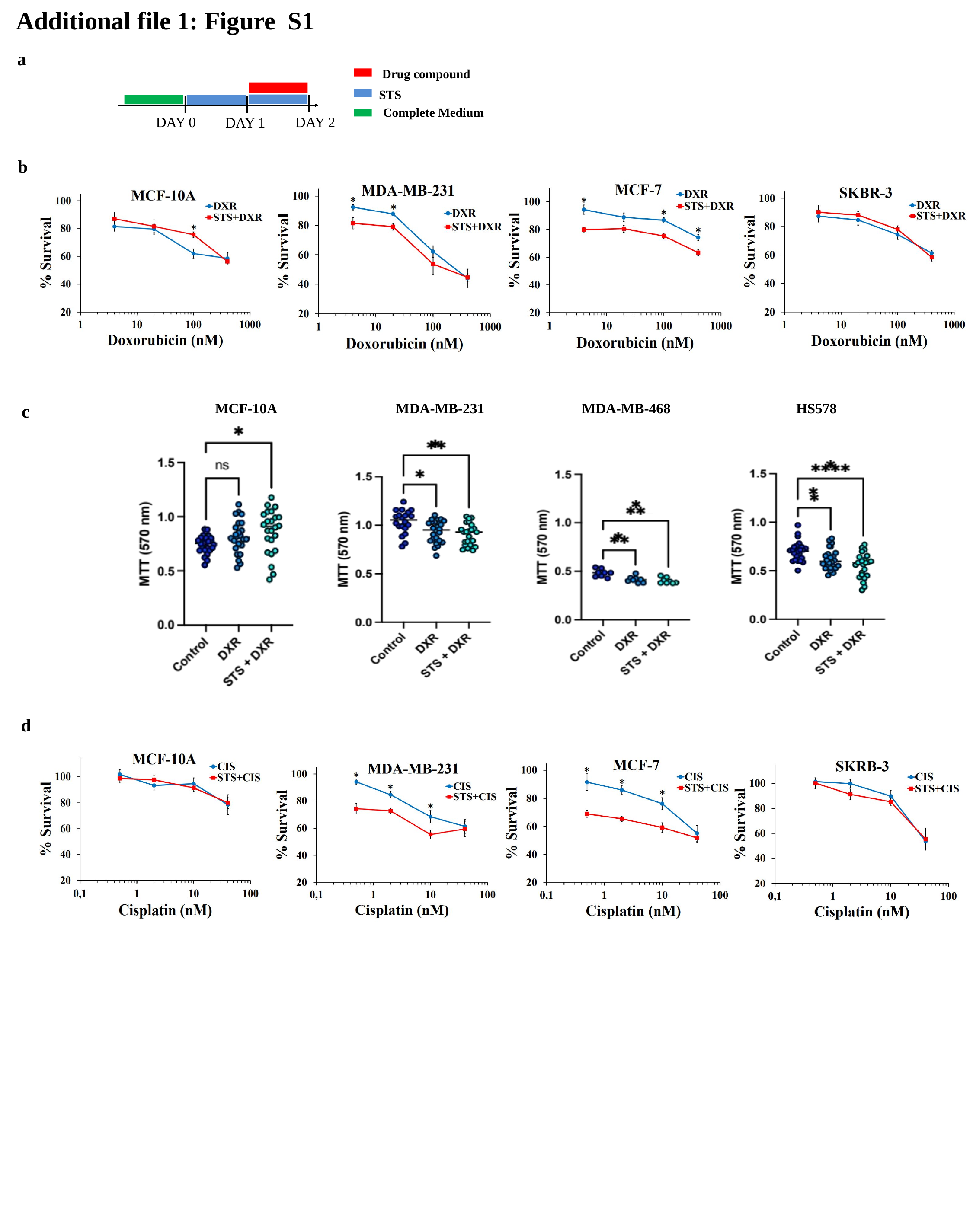

Additional file 1: Figure S1
a
Drug compound
STS
Complete Medium
DAY 0
DAY 2
DAY 1
b
MCF-10A
MDA-MB-231
MDA-MB-468
HS578
c
d
