# Supplementary material for: Short term starvation potentiates the efficacy of chemotherapy in triple negative breast cancer via metabolic reprogramming
Source: J Transl Med. 2023 Mar 3;21:169. doi: 10.1186/s12967-023-03935-9 (PMC9983166; doi:10.1186/s12967-023-03935-9)
Supplement: Supplementary file 2 — Additional file 2: Fig. S2. Differential accumulation of reactive oxygen species in malignant and near normal epithelial breast cells after starvation. a. Assessment of intracellular ROS production; fold change of intracellular ROS production compared to control in breast cancer cell lines upon STS, DXR or combined treatment (STS+DXR). Data are presented as mean fluorescent intensity +SD. *P ≤ 0.05. b. MTT survival assay after 48 h treatment with combined STS+DXR in absence or presence of NAC (4mM) in MDA-MB-231 cells. Data are presented as mean survival percentage. *P ≤ 0.05. c. Transcriptional analysis of NRF2 coupled with the downstream transcriptional targets NQO1 and TXNRD1 in the presented conditions in MCF-10A, MDA-MB-231, MDA-MB-468 and HS578 cell lines. Data are presented as mean expression values. *P ≤ 0.05. d. MTT survival assays after 48 h treatment with STS with or without addition of ΤΗ1579 in near normal MCF-10A cells and the indicated breast cancer cell lines. Data are presented as mean survival percentage. *P ≤ 0.05. e. γH2AΧ immunofluorescence staining in MCF-10A, MDA-MB-231, MDA-MB-468 and HS578 in Control (CTRL), DXR, STS and STS+DXR. Nuclei counterstained with DAPI. Scale bar, 5μm. Data are presented as mean of the number of foci per cell. *P ≤ 0.05. [file 12967_2023_3935_MOESM2_ESM.ppt]

## Slide 1
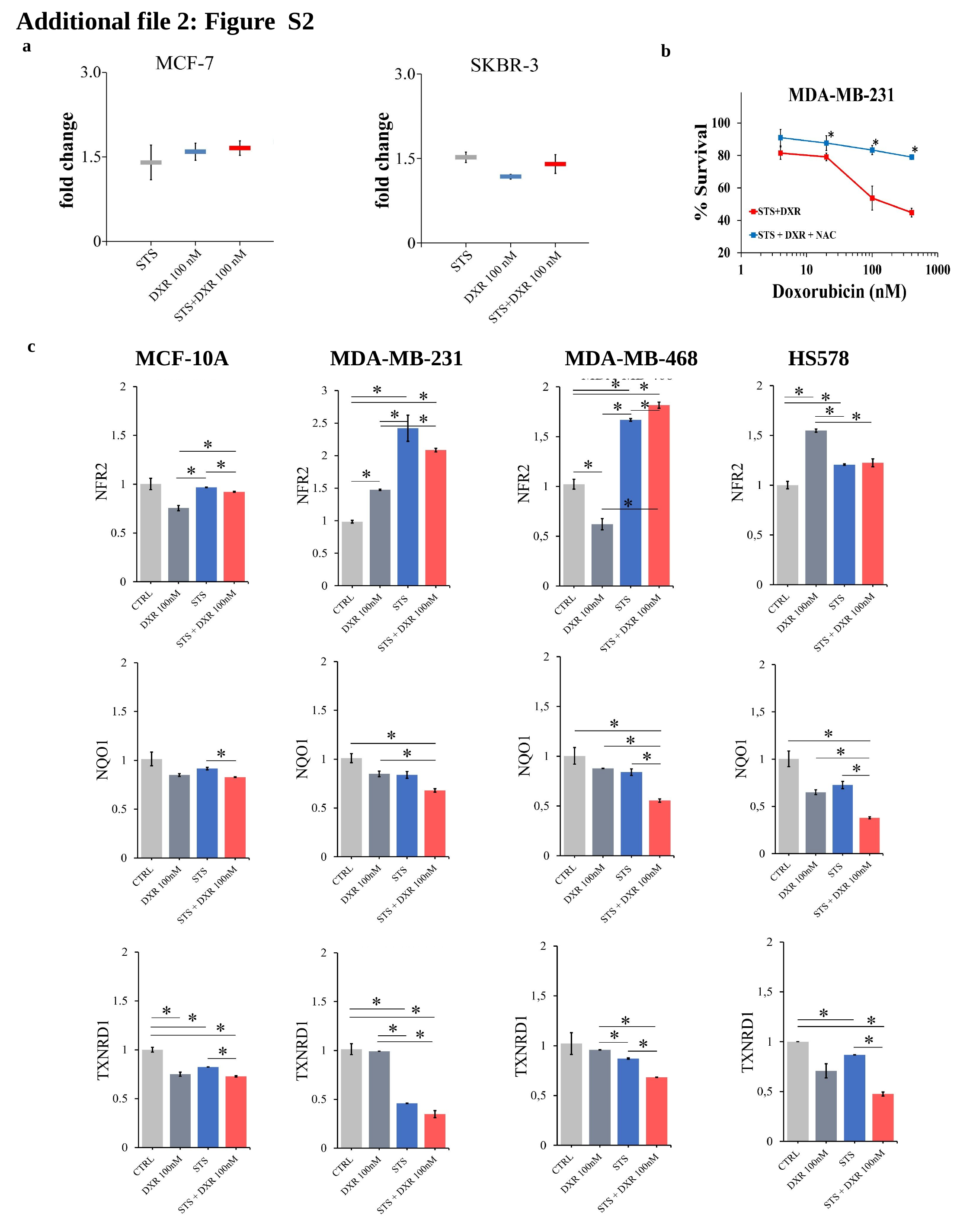

Additional file 2: Figure S2
a
b
c
MCF-10A
MDA-MB-231
MDA-MB-468
HS578

## Slide 2
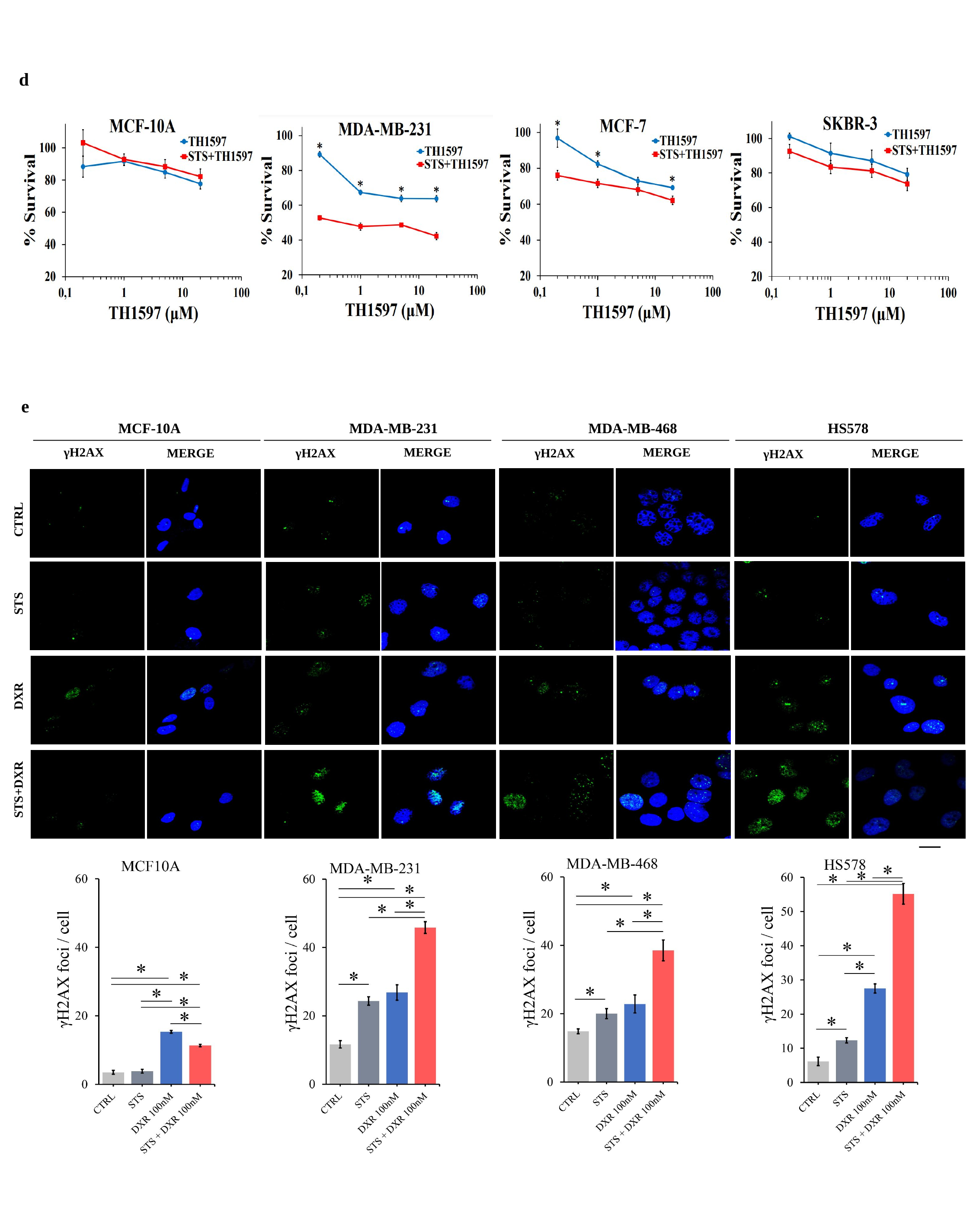

d
e
MCF-10A
MDA-MB-231
MDA-MB-468
HS578
MERGE
MERGE
MERGE
MERGE
γΗ2ΑΧ
γΗ2ΑΧ
γΗ2ΑΧ
γΗ2ΑΧ
CTRL
STS
DXR
STS+DXR
