# Supplementary material for: Short term starvation potentiates the efficacy of chemotherapy in triple negative breast cancer via metabolic reprogramming
Source: J Transl Med. 2023 Mar 3;21:169. doi: 10.1186/s12967-023-03935-9 (PMC9983166; doi:10.1186/s12967-023-03935-9)
Supplement: Supplementary file 3 — Additional file 3: Fig. S3. Combined treatment decreases oxidative phosphorylation and increases glycolytic reserve in highly aggressive TNBC cells. a. Oxygen Consumption Rate (OCR) measurements in MCF-10A and MDA-MB-231 cells treated with DXR alone or in combination with STS, using Seahorse Analyzer. Combination treatment selectively reduces basal respiration (corresponding to basal OCR), maximal respiration (corresponding to FCCP response) and spare respiratory capacity (the difference between maximal respiration and basal respiration). b. Extracellular Acidification Rate (ECAR) measurements in MCF-10A and MDA-MB-231 cells treated with DXR alone or in combination with STS, using Seahorse Analyzer. Combination treatment selectively increases glycolytic capacity, glycolytic reserve and non-glycolytic acidification in breast cancer cells. Data are presented as mean ECAR ±SD. Ns: non-significant; *P ≤ 0.05. [file 12967_2023_3935_MOESM3_ESM.ppt]

## Slide 1
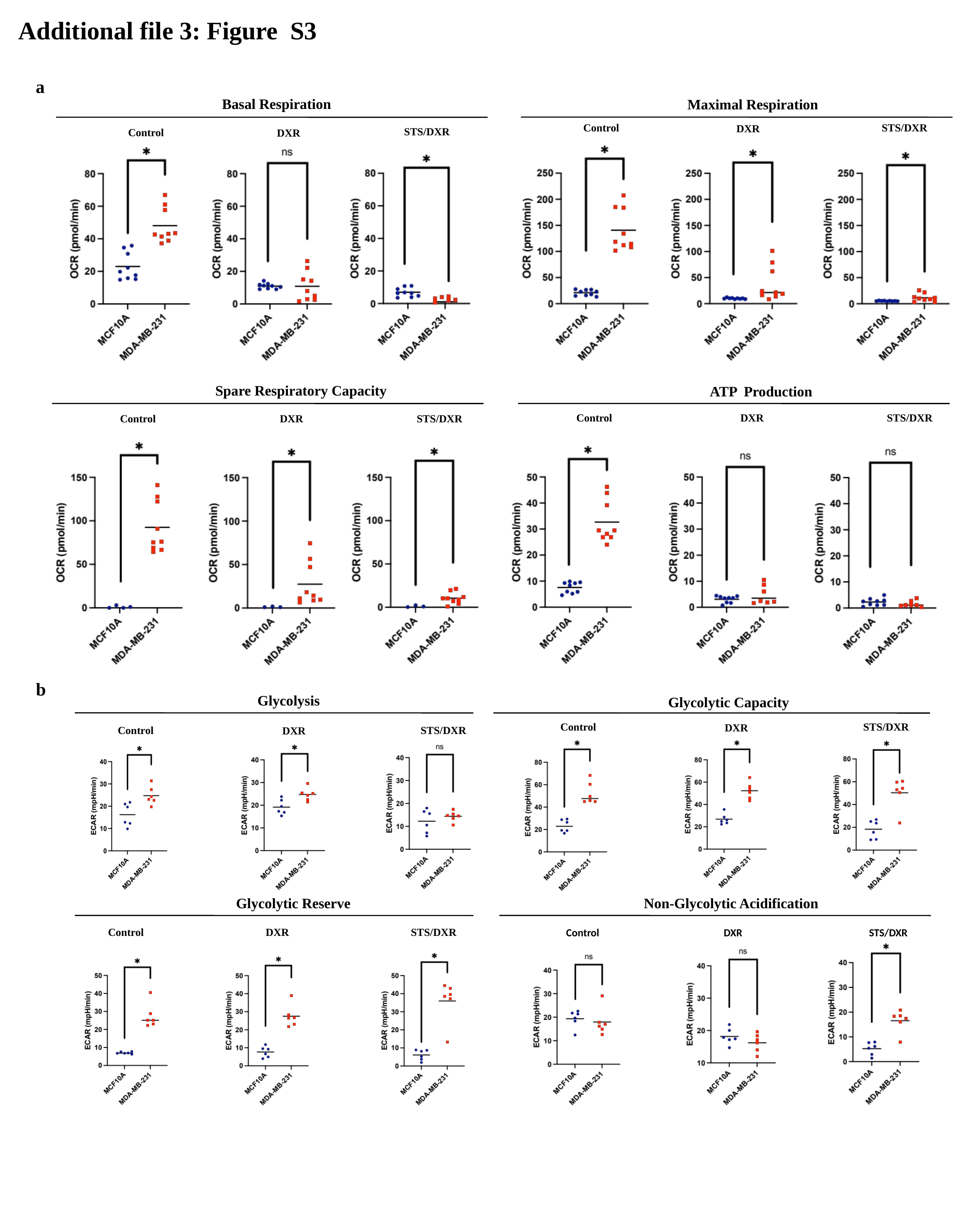

Additional file 3: Figure S3
a
Basal Respiration
Maximal Respiration
Control
STS/DXR
DXR
STS/DXR
Control
DXR
Spare Respiratory Capacity
ATP Production
Control
DXR
STS/DXR
Control
DXR
STS/DXR
b
Glycolysis
Control
STS/DXR
DXR
Glycolytic Capacity
Control
STS/DXR
DXR
Glycolytic Reserve
Non-Glycolytic Acidification
Control
DXR
STS/DXR
Control
DXR
STS/DXR
