# Supplementary material for: Short term starvation potentiates the efficacy of chemotherapy in triple negative breast cancer via metabolic reprogramming
Source: J Transl Med. 2023 Mar 3;21:169. doi: 10.1186/s12967-023-03935-9 (PMC9983166; doi:10.1186/s12967-023-03935-9)
Supplement: Supplementary file 4 — Additional file 4: Fig. S4. Combined treatment induces differential metabolic reprogramming. a. Intracellular metabolites profile in MCF-10A and MDA-MB-231 treated with DXR or STS+DXR. Cells were harvested on day 0, day 1 and day 2. *P ≤ 0.05. b. Expression of ATP5A in triple negative cell lines MDA-MB-468 and HS578 upon DXR or STS+DXR assessed by immunofluorescence analysis. Left panel: representative micrograph where ATP5A is shown in green, nuclei were counterstained with DAPI (blue). Scale bar, 20 μm. Right panel: Quantification, where data are presented as mean corrected total cell fluorescence (CTCF) ±SD. *P ≤ 0.05. c. Expression of ATP5A in MCF-7 and SKBR-3 cell lines upon DXR or STS+DXR assessed by immunofluorescence analysis. Left panel: representative micrograph where ATP5A is shown in green, nuclei were counterstained with DAPI (blue). Scale bar, 20 μm. Right panel: Quantification, where data are presented as mean corrected total cell fluorescence (CTCF) ±SD. *P ≤ 0.05. #Statistical trend. [file 12967_2023_3935_MOESM4_ESM.ppt]

## Slide 1
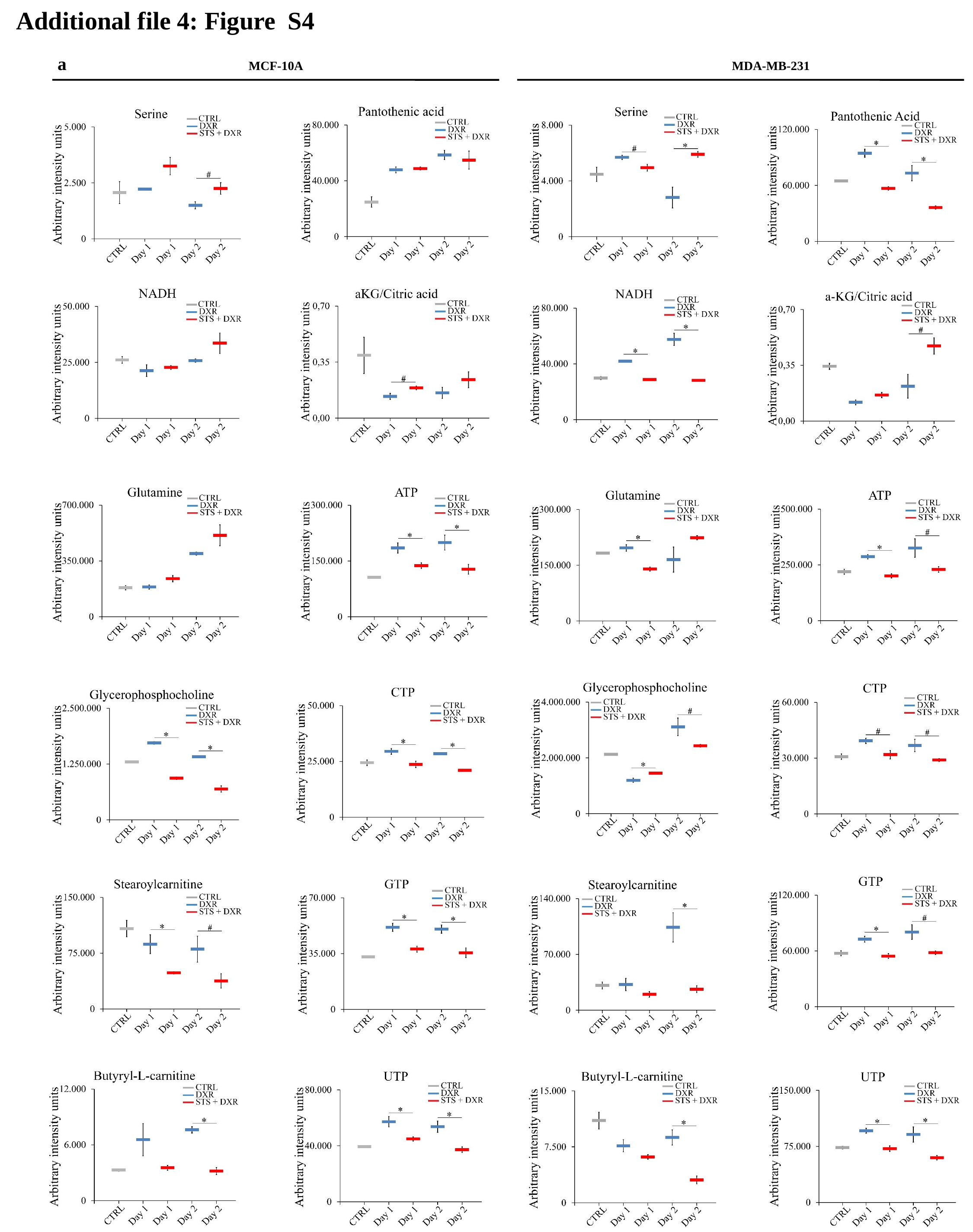

Additional file 4: Figure S4
a
MCF-10A
MDA-MB-231

## Slide 2
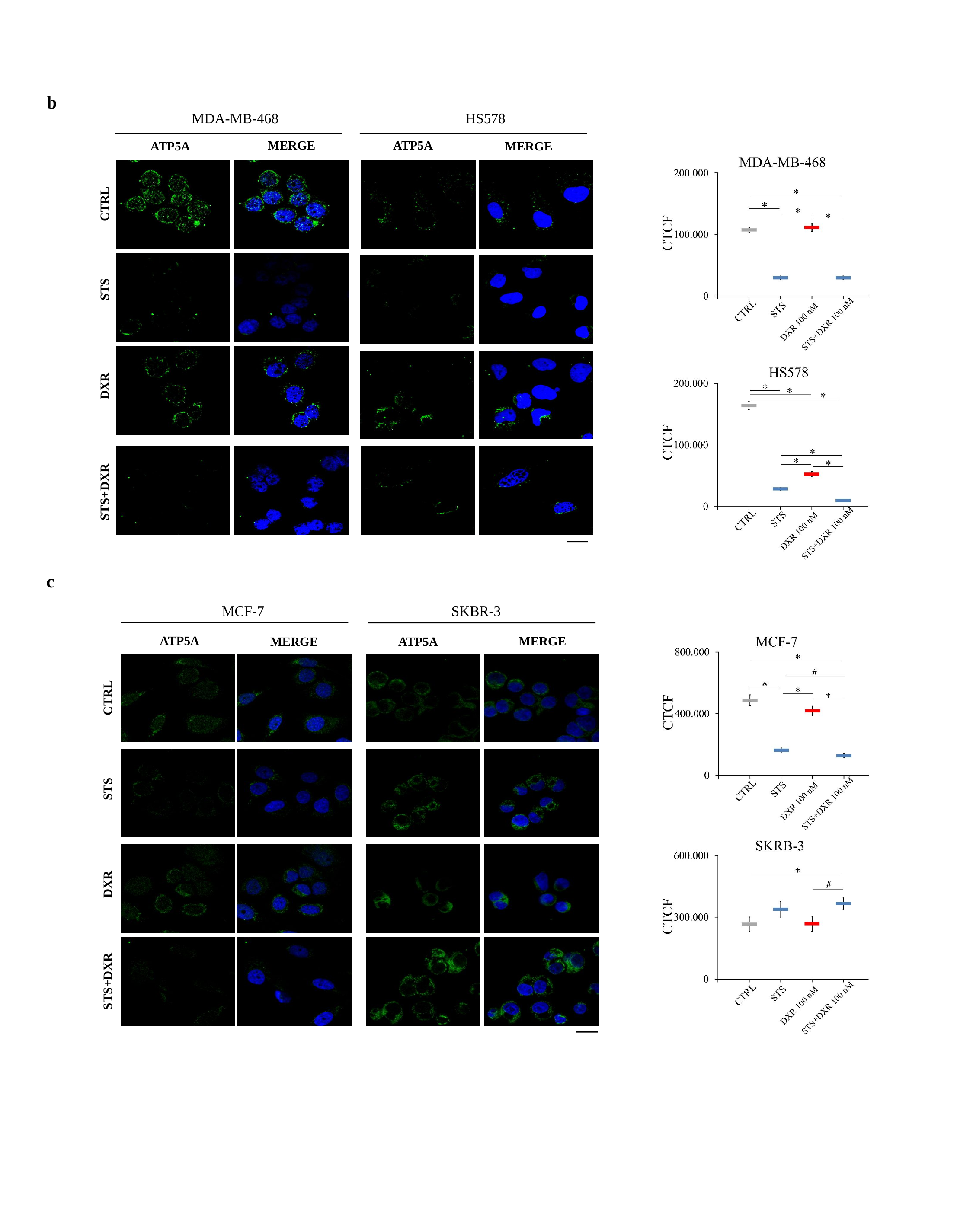

b
MDA-MB-468
HS578
MERGE
MERGE
ATP5A
ATP5A
CTRL
STS
DXR
STS+DXR
c
MCF-7
SKBR-3
MERGE
MERGE
ATP5A
ATP5A
CTRL
STS
DXR
STS+DXR
