# Supplementary material for: Short term starvation potentiates the efficacy of chemotherapy in triple negative breast cancer via metabolic reprogramming
Source: J Transl Med. 2023 Mar 3;21:169. doi: 10.1186/s12967-023-03935-9 (PMC9983166; doi:10.1186/s12967-023-03935-9)
Supplement: Supplementary file 5 — Additional file 5: Fig. S5. ATP5A1 silencing mimicks the effect of combined STS+DXR treatment in TNBC cells - FOXO1 silencing eliminate the selective sensitivity of TNBC cells to STS+DXR treatment. a. Transcriptional levels of ATP5A1 upon ATP5A1 silencing in MCF10A and MDA-MB-231. Data are presented as mean expression values. *P ≤ 0.05. b. Transcriptional levels of FOXO1 upon FOXO1 silencing in MCF10A and MDA-MB-231. Data are presented as mean expression values. *P ≤ 0.05. c. γH2AΧ immunofluorescence staining foci per cell in MCF-10A and MDA-MB-231 cells with or without ATP5A1 silencing. Nuclei counterstained with DAPI. Scale bar, 5μm. Data are presented as mean of the number of foci per cell. *P ≤ 0.05. d. γH2AΧ immunofluorescence staining foci per cell in STS+DXR treated MCF-10A and MDA-MB-231 cells with or without FOXO1 silencing. Nuclei counterstained with DAPI. Scale bar, 5μm. Data are presented as mean of the number of foci per cell. *P ≤ 0.05. e. Transcriptional analysis of NRF2 coupled with the downstream targets NQO1 and TXNRD1 in MCF-10A and MDA-MB-231 cell lines with or without ATP5A1 silencing. Data are presented as mean expression values. *P ≤ 0.05. f. Transcriptional analysis of NRF2 coupled with the downstream targets NQO1 and TXNRD1 in STS+DXR treated MCF-10A and MDA-MB-231 cell lines with or without FOXO1 silencing. Data are presented as mean expression values. *P ≤ 0.05. [file 12967_2023_3935_MOESM5_ESM.ppt]

## Slide 1
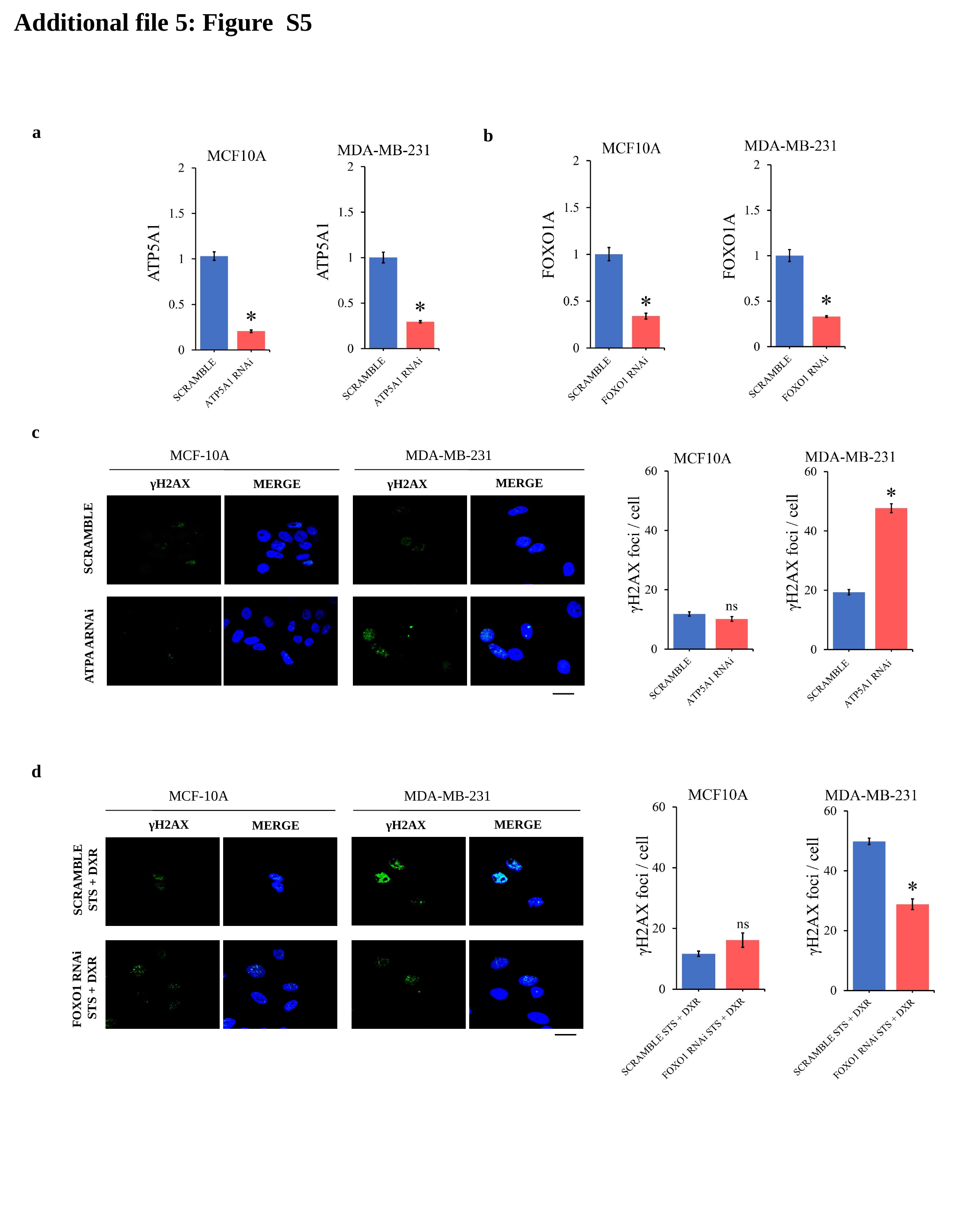

Additional file 5: Figure S5
a
b
c
MCF-10Α
MDA-MB-231
MERGE
MERGE
γΗ2ΑΧ
γΗ2ΑΧ
SCRAMBLE
ATPA ARNAi
d
MCF-10Α
MDA-MB-231
MERGE
MERGE
γΗ2ΑΧ
γΗ2ΑΧ
SCRAMBLE STS + DXR
FOXO1 RNAi STS + DXR

## Slide 2
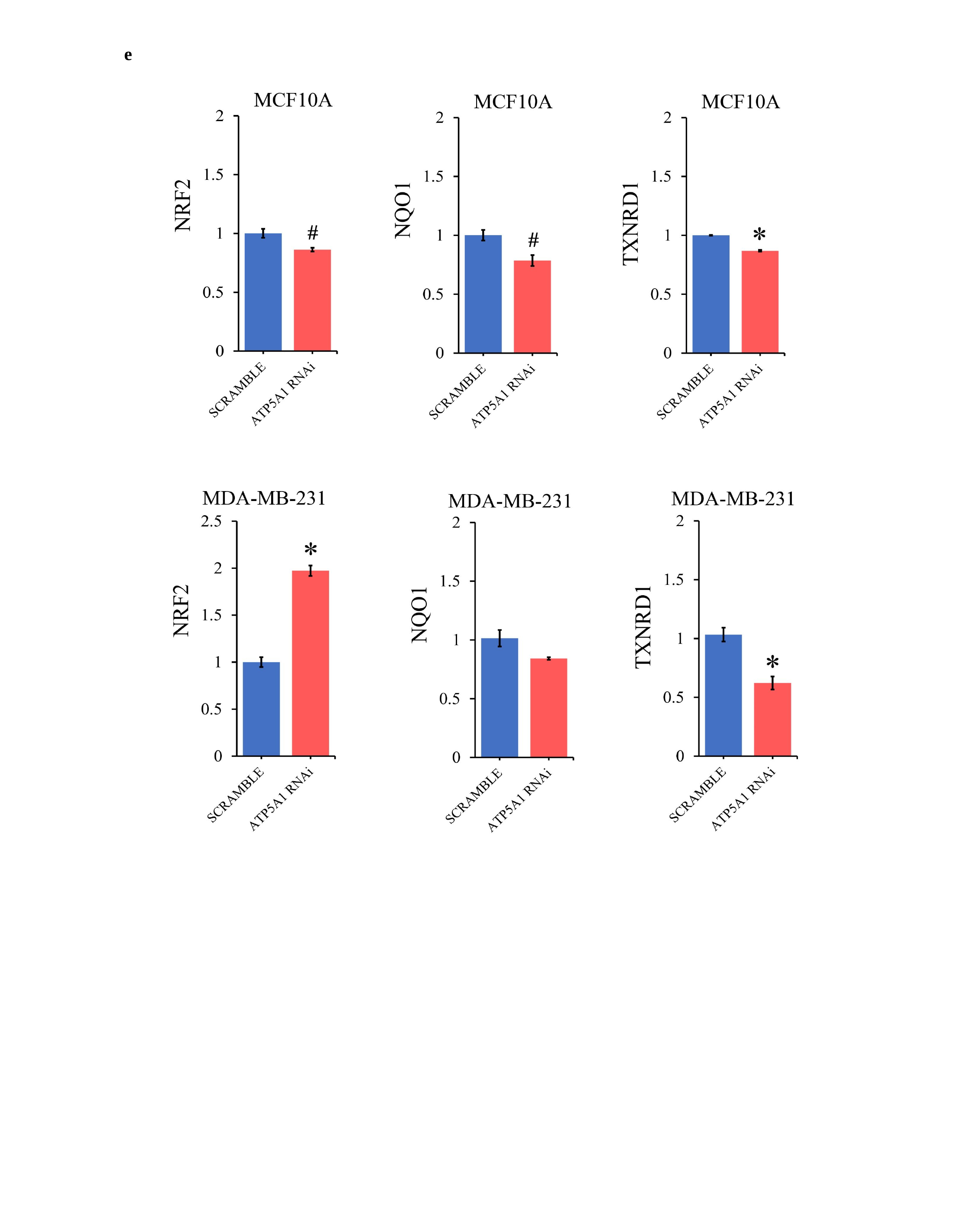

e

## Slide 3
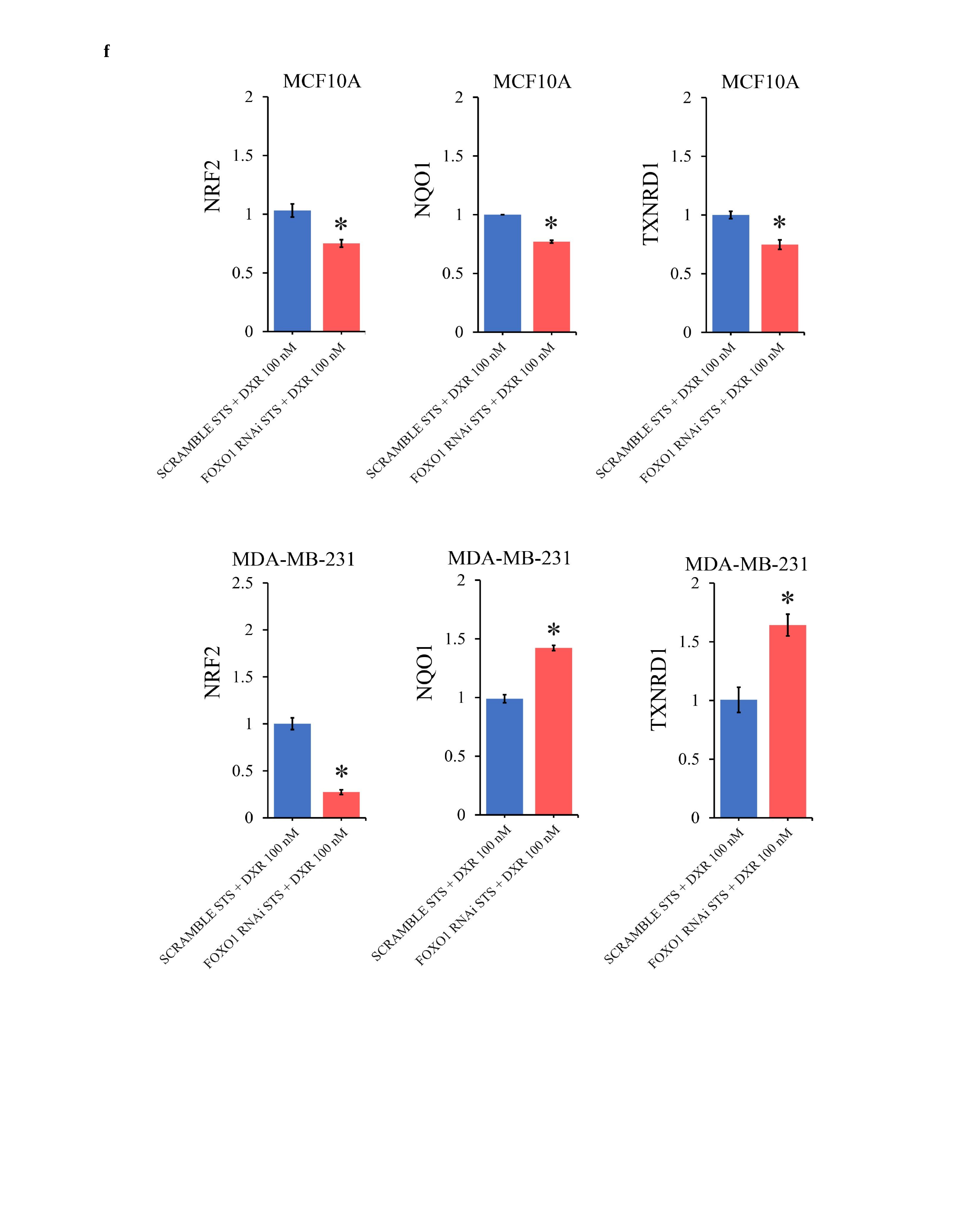

f
