# Supplementary material for: Short term starvation potentiates the efficacy of chemotherapy in triple negative breast cancer via metabolic reprogramming
Source: J Transl Med. 2023 Mar 3;21:169. doi: 10.1186/s12967-023-03935-9 (PMC9983166; doi:10.1186/s12967-023-03935-9)
Supplement: Supplementary file 6 — Additional file 6: Fig. S6. Caloric restriction combined with chemotherapy retards tumor growth and suppresses metastatic potential in a triple-negative breast cancer mouse model. a. MTT survival assays of 4T1 mouse breast cancer cells treated with DXR alone or in combination with STS (STS+DXR). Data are presented as mean survival percentage. *P ≤ 0.05. b. Assessment of intracellular ROS production. Data are presented as mean of the fold change ±SD of intracellular ROS production in cells upon STS, DXR or combined STS+DXR. *P ≤ 0.05. c. Levels of key biochemical markers measured in serum of mice injected with 4T1 cells and treated with CR, DXR or combined CR+DXR treatment. Markers were measured in three mice from each treatment group. Data are presented as mean ±SD. One Way Anova-Kruskal Wallis test. d. Primary whole-tumor pathological specimen collected from control mice and mice treated with CR+/-DXR and euthanized at day 35. e. Mitochondria from tumor derived from control mice and mice treated with CR+/-DXR were isolated and evaluated with oxygraphy for respiration parameters. Data are presented as mean ±SD. #P<0.01 (statistical trend); *P ≤ 0.05. [file 12967_2023_3935_MOESM6_ESM.ppt]

## Slide 1
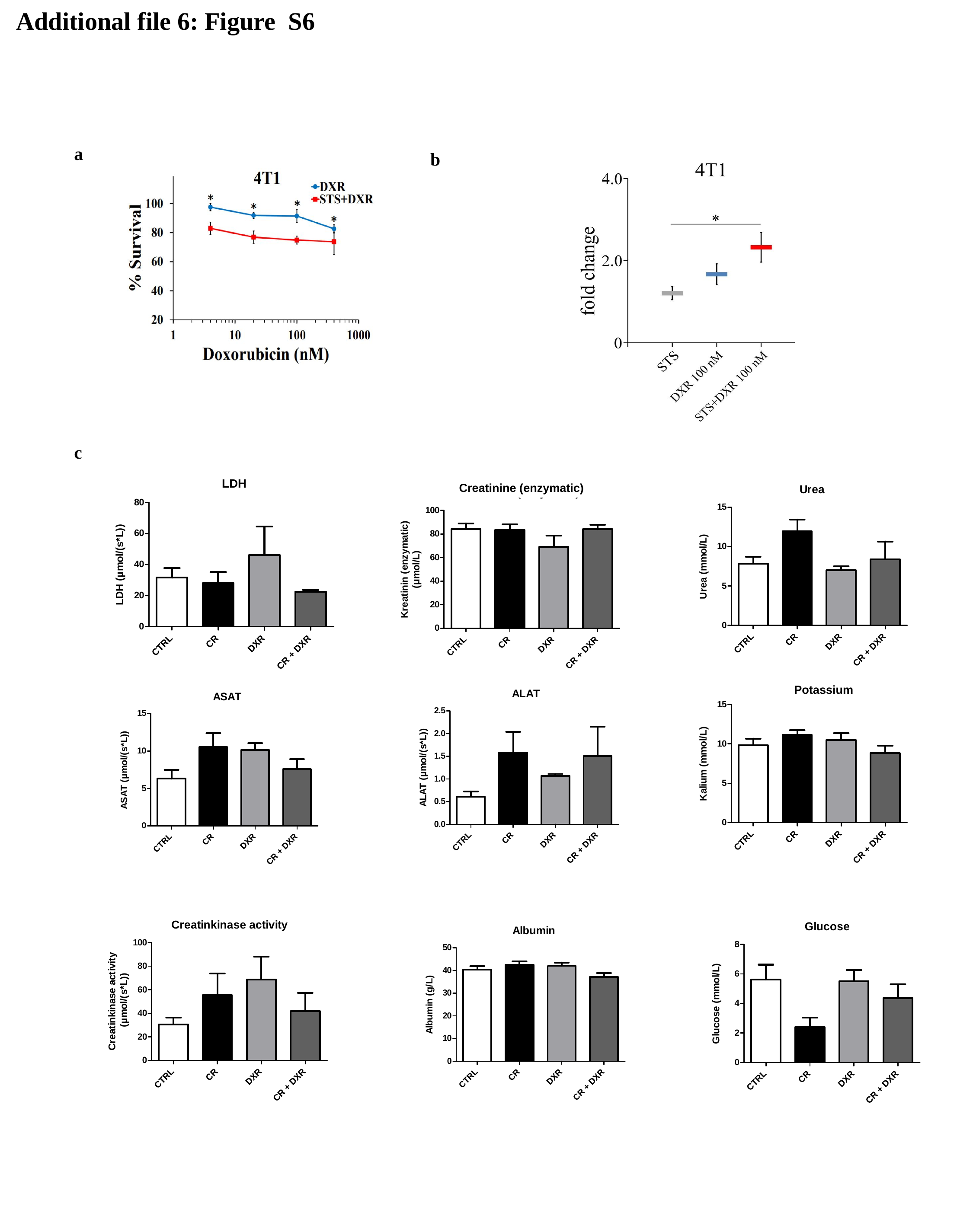

Additional file 6: Figure S6
a
b
c
Creatinine (enzymatic)
Potassium

## Slide 2
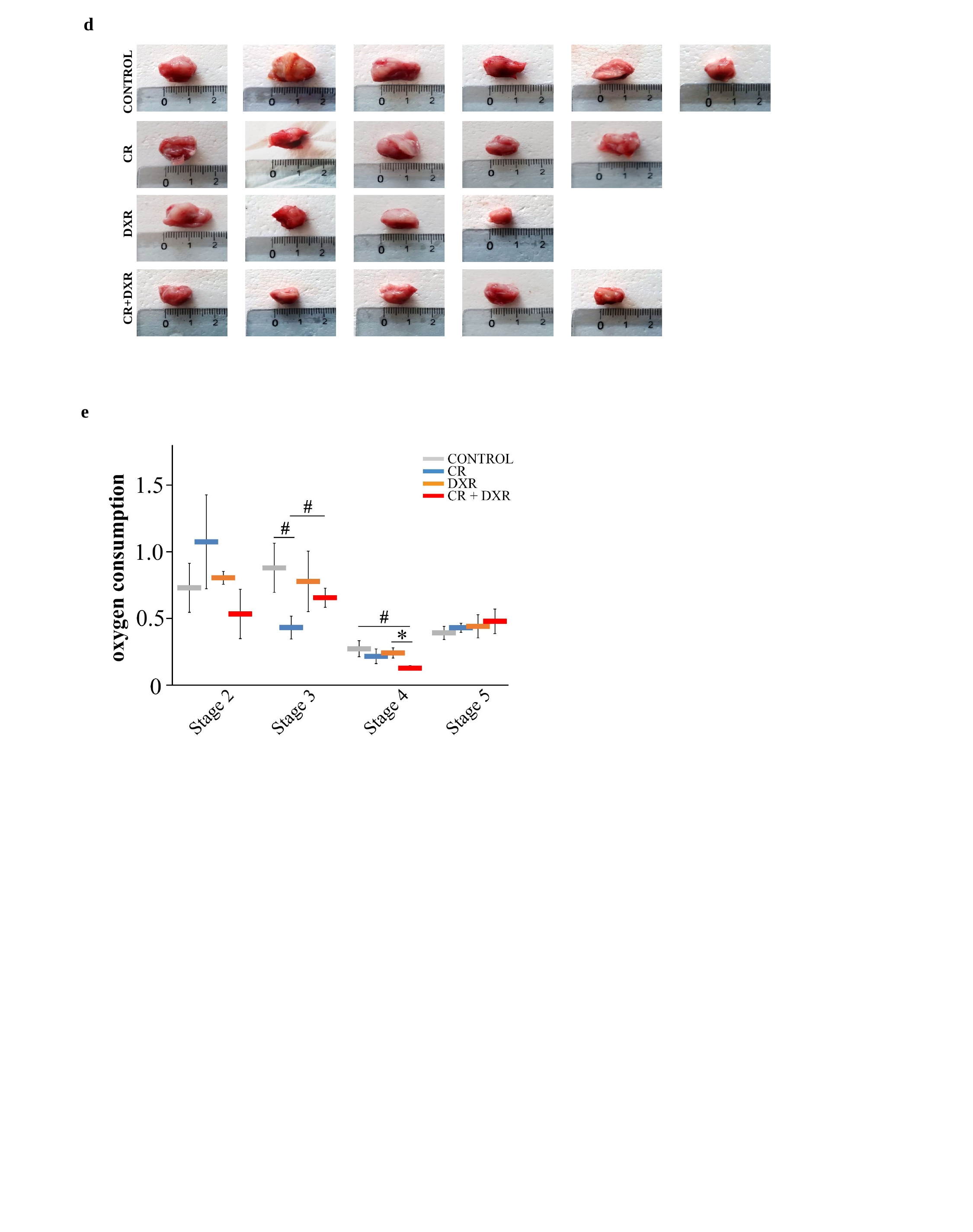

d
CR+DXR DXR CR CONTROL
e
