# Supplementary figures and images for: Short term starvation potentiates the efficacy of chemotherapy in triple negative breast cancer via metabolic reprogramming
Source: J Transl Med. 2023 Mar 3;21:169. doi: 10.1186/s12967-023-03935-9 (PMC9983166; doi:10.1186/s12967-023-03935-9)

## Slide 1
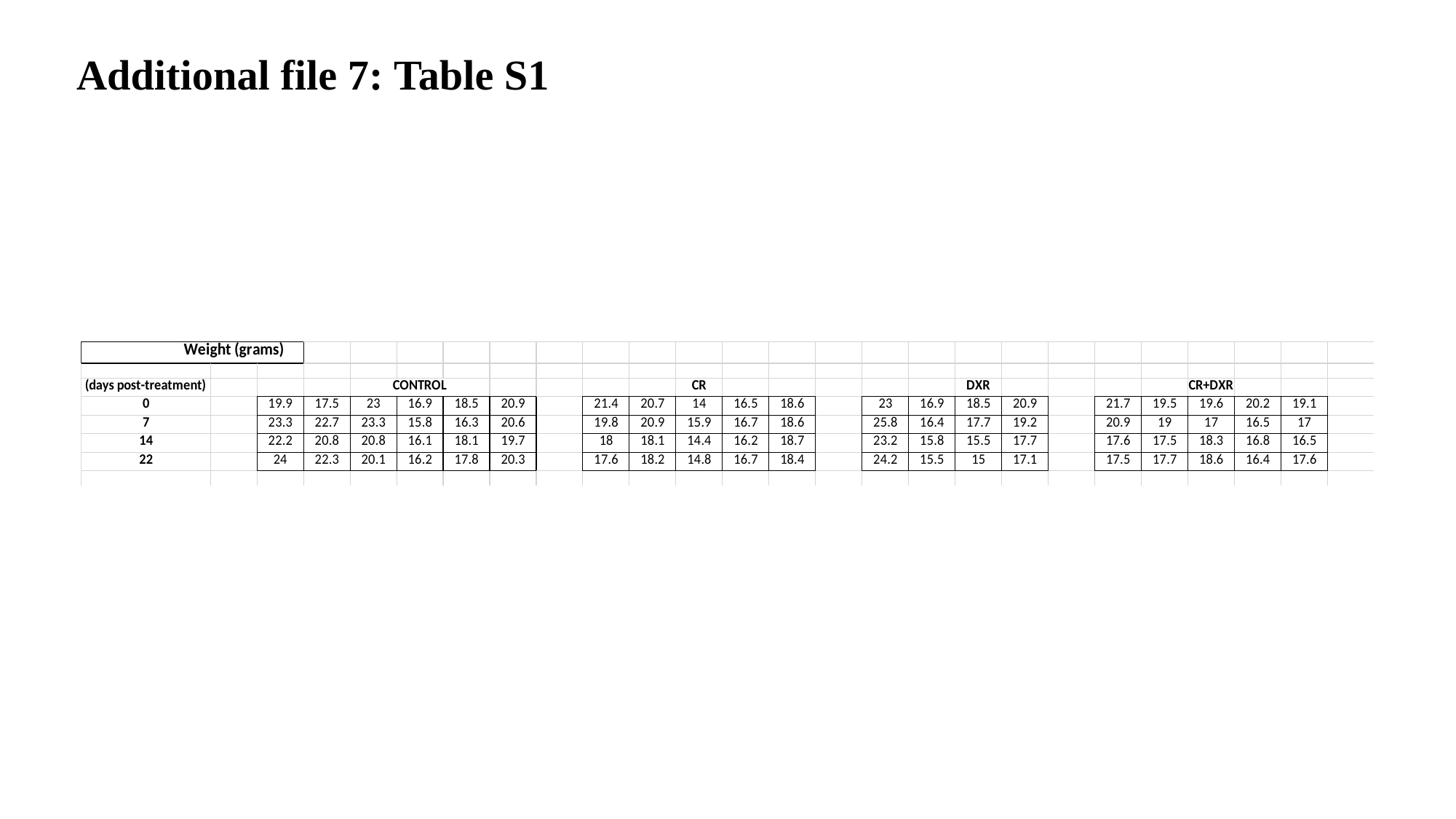

Additional file 7: Table S1

Supplement: Supplementary file 7 — Additional file 7: Table. S1. Weight (in grams) monitoring in control, caloric restricted (CR), DXR treated and CR+DXR treated mice. [file 12967_2023_3935_MOESM7_ESM.pptx]
