# Supplementary material for: Short term starvation potentiates the efficacy of chemotherapy in triple negative breast cancer via metabolic reprogramming
Source: J Transl Med. 2023 Mar 3;21:169. doi: 10.1186/s12967-023-03935-9 (PMC9983166; doi:10.1186/s12967-023-03935-9)
Supplement: Supplementary file 8 — Additional file 8. Methods. [file 12967_2023_3935_MOESM8_ESM.docx]

**Additional information**

**Methods**

LC/MS Analysis.

Metabolic profiling by LC-MS was performed at the Swedish Metabolomics Center in Umeå, Sweden.

Sample Preparation: At day 0, day 1, day 2 during treatment, adherent growing cells were washed three times with ice-cold PBS while keeping the culturing plate on ice. Cells were harvested by scraping in 750 µL ice cold methanol and then stored at -80°C until further treatment. With samples on ice, one 3 mm tungsten carbide bead (QIAGEN) was added to each sample and cells were disrupted by shaking at 30 Hz for 2 minutes (Mixer Mill MM400, Retsch) using pre-chilled holding blocks (4°C). Samples were centrifuged at 14.000 RPM for 10 minutes at 4°C and then 200 µL supernatant was transferred into LC-MS vials (Chromacol, 03-FISV, Thermo Fisher, MA, USA). Samples were evaporated (miVac, GeneVac) at room temperature until dried and stored at -80°C until analysis.

A small aliquot per sample-type of the remaining respective supernatants were pooled and used to create quality control (QC) samples. MSMS analysis was run on the QC samples for identification purposes.

The samples were analyzed in batches according to a randomized run order on LC-MS.

LC-MS analysis: Before LCMS analysis the sample was re-suspended in 10 + 10 µL methanol and water containing internal standards (IS). Each batch of samples (sample-type) was first analyzed in positive mode. After all samples within a batch had been analyzed, the instrument was switched to negative mode and a second injection of each sample was performed.

The reversed phase chromatographic separation was performed on an Agilent 1290 Infinity UHPLC-system (Agilent Technologies, Waldbronn, Germany). Two μL of each sample were injected onto an Acquity UPLC HSS T3, 2.1 x 50 mm, 1.8 μm C18 column in combination with a 2.1 mm x 5 mm, 1.8 μm VanGuard precolumn (Waters Corporation, Milford, MA, USA) held at 40 °C. The gradient elution buffers were A (H2O, 0.1 % formic acid) and B (75/25 acetonitrile:2-propanol, 0.1 % formic acid), and the flow-rate was 0.5 mL min-1. The compounds were eluted with a linear gradient consisting of 0.1 - 10 % B over 2 minutes, B was increased to 99 % over 5 minutes and held at 99 % for 2 minutes; B was decreased to 0.1 % for 0.3 minutes and the flow-rate was increased to 0.8 mL min-1 for 0.5 minutes; these conditions were held for 0.9 minutes, after which the flow-rate was reduced to 0.5 mL min-1 for 0.1 minutes before the next injection. The HILIC chromatographic analysis was performed using a iHILIC-Fusion(+) column, 100x2.1 mm, 3.5 µM, 100 Å, from Hilicon AB (Umeå, Sweden). HILIC elution solvents were (A) H2O, 50 mM ammonium formate (B) 90:10 Acetonitrile:[H2O 50 mM ammonium formate], total concentration ammonium formate in B 5 mM. Chromatographic separation was achieved using the following linear gradient (flow rate 0.4 mL/min), min 0: 90% B; min 4: 85% B, min 5: 70% B, min 7: 55% B, min 10: 20% B, min 10.01: 90% B, min 15: 90% B.

The compounds were detected with an Agilent 6550 Q-TOF mass spectrometer equipped with a jet stream electrospray ion source operating in positive or negative ion mode. The settings were kept identical between the modes, with exception of the capillary voltage. A reference interface was connected for accurate mass measurements; the reference ions purine (4 μM) and HP-0921 (Hexakis (1H, 1H, 3H-tetrafluoropropoxy)phosphazine) (1 μM) were infused directly into the MS at a flow rate of 0.05 mL min-1 for internal calibration, and the monitored ions were purine m/z 121.05 and m/z 119.03632; HP-0921 m/z 922.0098 and m/z 966.000725 for positive and negative mode respectively. The gas temperature was set to 150°C, the drying gas flow to 16 L min-1 and the nebulizer pressure 35 psig. The sheath gas temp was set to 350°C and the sheath gas flow 11 L min-1. The capillary voltage was set to 4000 V in positive ion mode, and to 4000 V in negative ion mode. The nozzle voltage was 300 V. The fragmentor voltage was 380 V, the skimmer 45 V and the OCT 1 RF Vpp 750 V. The collision energy was set to 0 V. The m/z range was 70 - 1700, and data was collected in centroid mode with an acquisition rate of 4 scans s-1 (1977 transients/spectrum).

Data Analysis: For the LC-MS data, all data processing was performed using the Agilent Masshunter Profinder version B.10.00 (Agilent Technologies Inc., Santa Clara, CA, USA).

The processing was performed in a target fashion. For target processing, a pre-defined list of metabolites commonly found in plasma and serum were searched for using the Batch Targeted feature extraction in Masshunter Profinder. An-in-house LC-MS library built up by authentic standards run on the same system with the same chromatographic and mass-spec settings, were used for the targeted processing. The identification of the metabolites was based on MS, MSMS and retention time information.

Solvents: Methanol, HPLC-grade was obtained from Fischer Scientific (Waltham, MA, USA) Acetonitrile and 2-Propanol, both LC-MS grade was obtained from Merck KGaA (Darmstadt, Germany) H2O, Milli-Q. Reference and tuning standards: Purine, 4 μM, Agilent Technologies (Santa Clara, CA, USAhh) HP-0921 (Hexakis(1H, 1H, 3H-tetrafluoropropoxy)phosphazine), 1 μM, Agilent Technologies (Santa Clara, CA, USA) Calibrant, ESI-TOF, ESI-L Low Concentration Tuning Mix, Agilent Technologies (Santa Clara, CA, USA) HP-0321 (Hexamethoxyphosphazine), 0.1 mM, Agilent Technologies (Santa Clara, CA, USA). Stable isotopes internal standards: LC-MS internal standards: 13C9-Phenylalanine, 13C3-Caffeine, D4-Cholic acid, 13C9-Caffeic Acid and salicylic acid-D6 were obtained from Sigma (St. Louis, MO, USA).
